# Linking microsomal prostaglandin E Synthase-1/PGE-2 pathway with miR-15a and −186 expression: Novel mechanism of VEGF modulation in prostate cancer

**DOI:** 10.18632/oncotarget.10051

**Published:** 2016-06-14

**Authors:** Erika Terzuoli, Sandra Donnini, Federica Finetti, Gabriella Nesi, Donata Villari, Hiromi Hanaka, Olof Radmark, Antonio Giachetti, Marina Ziche

**Affiliations:** ^1^ Department of Life Sciences, University of Siena, 53100, Siena, Italy; ^2^ Istituto Toscano Tumori (ITT), 50136, Florence, Italy; ^3^ Department of Surgery and Translational Medicine, University of Florence, 50136, Florence, Italy; ^4^ Department of Clinical and Experimental Medicine, University of Florence, 50136, Florence, Italy; ^5^ Department of Medical Biochemistry and Biophysics, Karolinska Institutet, S-171 77, Stockholm, Sweden

**Keywords:** miR-186, mPGES-1/PGE-2, VEGF, prostate cancer, tumor angiogenesis

## Abstract

Prostaglandin E-2 (PGE-2) promotes tumor angiogenesis via paracrine secretion of pro-angiogenic growth factors, such as vascular endothelial growth factor (VEGF). Since miRNAs regulate several cell processes, including angiogenesis, we sought to determine whether they would influence PGE-2-induced VEGF. We compared DU145 and PC3 prostate cancer cells bearing the mPGES-1 enzyme (mPGES-1^+/+^) and producing PGE-2, with those in which the enzyme was silenced or deleted (mPGES-1^−/−^). We demonstrated that mPGES-1/PGE-2 signaling decreased Dicer expression and miRNA biogenesis. Genome-wide sequencing of miRNAs revealed that miR-15a and miR-186, associated with expression of VEGF and hypoxia inducible factor-1α (HIF-1α), were down-regulated in mPGES-1^+/+^ cells. As a consequence, mPGES-1^+/+^ tumor cells expressed high levels of VEGF and HIF-1α, induced endothelial cells activation and formed highly vascularized tumors. Mir-186 mimic inhibited VEGF expression in mPGES-1^+/+^ tumor xenografts and reduced tumor growth. In human prostate cancer specimens, mPGES-1 was over-expressed in tumors with high Gleason score, elevated expression of VEGF and HIF-1α, high microvessel density and decreased expression of Dicer, miR15a and miR-186. Thus, clear evidence for regulating miRNA processing and VEGF output by intrinsic PGE-2 production provides a means to distinguish between aggressive and indolent prostate tumors and suggests a potential target for controlling tumor progression.

## INTRODUCTION

Like other solid tumors, prostate cancer promotes angiogenesis to support its survival and inherent propensity to colonize other tissues. Many reports have described the close relationship between tumor and endothelium, as well as documenting the existence of a pool of tumor-derived angiocrine factors that induce vessel development, including vascular endothelial growth factor (VEGFA) [1]. Similar principles govern angiogenesis in prostate cancer, as VEGF and other factors secreted by tumors have been reported to promote neovascularization [2, 3]. Although, angiogenesis is often regarded as a distinguishing feature of aggressive versus indolent phenotype [4], its relevance for prostate cancer progression has been questioned on the basis of lack of efficacy of antiangiogenic agents [5, 6]. The robust tumorigenic drive of DU145 prostate cancer cells has been associated with constitutive over-expression of microsomal prostaglandin E synthase-1 (mPGES-1) and higher output of PGE-2 [7], which, in turn, impinges on several pro-tumorigenic pathways such as EGFR and Wnt [8–13].

Another layer of regulation for prostate cancer progression is the recently recognized role of miRNA expression, which appears to control several key processes of tumor biology [14]. In this work we sought to determine whether mPGES-1/PGE-2 signaling influence miRNA expression and whether these miRNAs might be involved in VEGF expression *in vitro* and tumor angiogenesis *in vivo*. By comparing DU145 and PC3 prostate cancer models bearing the mPGES-1 functional enzyme (transfected with scrambled control shRNA or CRISPR/Cas9 control, i.e. mPGES-1^+/+^ cells) with those in which the enzyme is silenced or deleted (knocked down or knocked out, i.e. mPGES-1^−/−^ cells), we provide evidence that tumor-derived PGE-2 represses miRNA biogenesis, inhibiting Dicer expression by an autocrine mechanism. Genome-wide sequencing of miRNAs in mPGES-1^+/+^ compared to mPGES-1^−/−^ cells revealed repression of miR-15a and miR-186, both associated with VEGF expression. Immunohistochemical analysis of mPGES-1 expression revealed that the enzyme was strongly expressed in human tumors with high Gleason score, VEGF, and HIF-1α as well as with microvessel density, and low expression of Dicer, miR15a and miR-186. Thus it appears that mPGES-1/PGE-2 signaling increases VEGF expression in prostate cancer by inhibiting the miRNA processing.

## RESULTS

### mPGES-1 expression is associated with tumor growth, VEGF expression and vessel density

**Figure 1:**
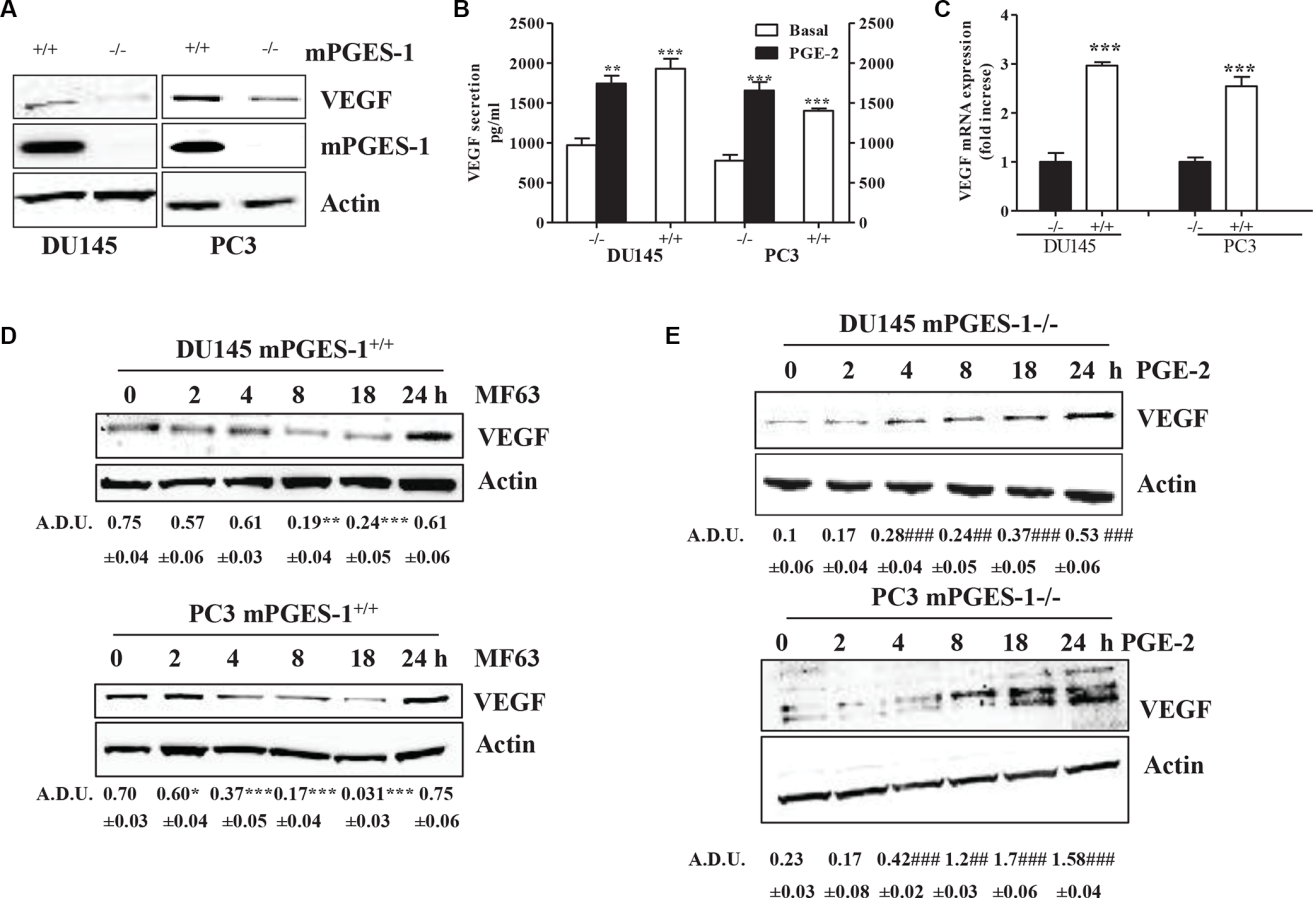
mPGES-1 increases VEGF expression (**A**) Western blot analysis of VEGF and mPGES-1 expression in DU145 and PC3 mPGES-1^+/+^ and in mPGES-1^−/−^ cells exposed to 10% FBS (48 h). b-actin was used to normalize loading; *N* = 3. (**B**) ELISA for VEGF in DU145 and PC3 mPGES-1^+/+^ and in mPGES-1^−/−^ cells exposed to FBS (1%, 48 h) or to PGE-2 (1 μM, 48 h). DU145 and PC3 mPGES-1^−/−^ were obtained by shRNA or CRISP/Cas9 transfection, respectively (see also Materials and Methods for details). ***P < 0.001, **p < 0.01 vs. mPGES-1^−/−^ cells. (**C**) VEGF mRNA expression in DU145 and PC3 mPGES-1^+/+^ and in mPGES-1^−/−^ cells exposed to 10% FBS (48 h). Data are reported as fold increase of mPGES-1^+/+^ vs. mPGES-1^−/−^ cells. ***P < 0.001 vs. mPGES-1^−/−^ cells. (**D**) VEGF protein expression in DU145 and PC3 mPGES-1^+/+^ exposed to MF63 (10 μM). (**E**) VEGF protein expression in DU145 and PC3 mPGES-1^−/−^ exposed to PGE-2 (1 μM). b-actin was used to normalize loading. *N* = 3. Data expressed in A.D.U. (arbitrary density unit) and as mean ± SD. ***P < 0.001 vs. untreated cells.

We investigated how prostate cancer cell-derived PGE-2 controls VEGF expression and tumor angiogenesis in DU145 and PC3 cells. VEGF expression/levels were up-regulated in mPGES-1^+/+^ compared to mPGES-1^−/−^ cells, as measured by QPCR western blot and ELISA (Figure 1A, 1B, 1C). When mPGES-1 was silenced, VEGF expression decreased. MF63 (10 μM), a reversible mPGES-1 inhibitor, significantly decreased VEGF expression in mPGES-1^+/+^ cells with maximum effect at 8–18 h (Figure 1D), while exogenous PGE-2 (1 μM) increased VEGF release and expression in mPGES-1^−/−^ cells time-dependently (Figure 1B, 1E).

**Figure 2:**
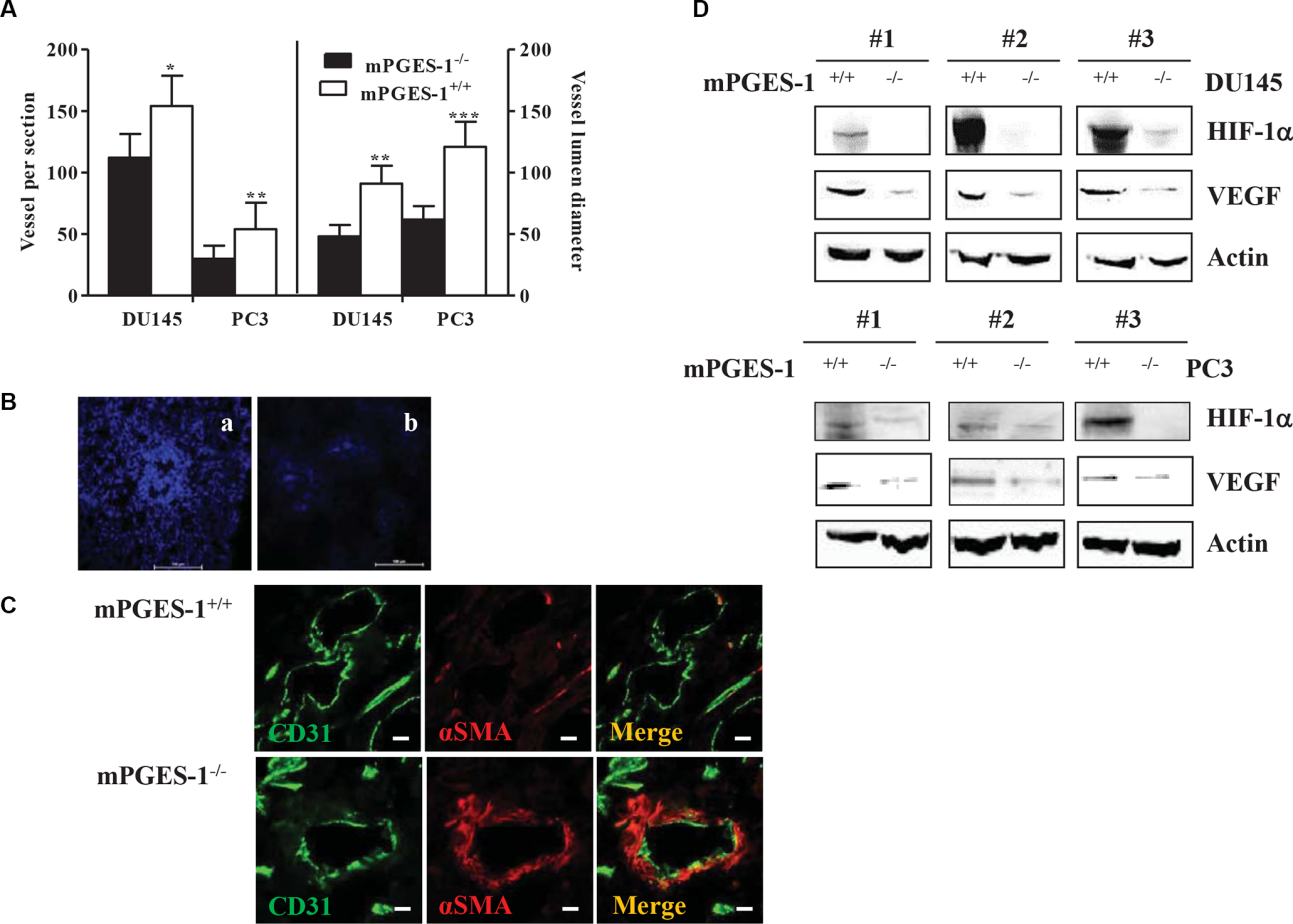
mPGES-1 induces tumor angiogenesis *in vivo* (**A**) Vessel number and size in DU145 and PC3 mPGES-1^+/+^ and in mPGES-1^−/−^ tumors. Quantification of human CD31 and vessel lumen was performed counting ten random fields/sections per slide; each slide had five sections. Data represents number of vessels counted per section (left) or vessel diameter (μm) in tumors (right). ***P < 0.001, **P < 0.01;*P < 0.05 vs. mPGES-1^−/−^. (**B**) Images of vessel perfusion, evaluated by Hoechst 32224 staining (blue), in mPGES-1^+/+^ (A) or mPGES-1^−/−^ tumors (B). Scale bars = 100 μm. Images taken at 10X magnification. (**C**) Images of double-immunostaining for CD31 (green), αSMA (red) and merge in tumor sections from mPGES-1^+/+^ (top) or mPGES-1^−/−^ group (bottom). Scale bars = 160 μm. Images obtained with confocal microscopy at 60X magnification. (**D**) HIF-1a and VEGF protein expression in tumors from DU145 and PC3 mPGES-1^+/+^ or mPGES-1^−/−^ mice. b-actin was used to normalize loading. *N* = 3.

In mouse xenograft of DU145 and PC3, mPGES-1^+/+^ tumor size was significantly higher than mPGES-1^−/−^ tumor size (Supplementary Figure S1A, S1B). mPGES-1^+/+^ tumors displayed higher vessel density and increased luminal size (Figure 2A left and right graph, respectively). Hoechst 33342 diffusion in mPGES-1^+/+^ tumors exhibited an intense and diffused perfusion, indicative of an abnormal vasculature (Figure 2B, panel A), while mPGES-1^−/−^ tumors had a reduced vascular perfusion around the vessels (Figure 2B, panel B). Moreover, mPGES-1/PGE-2 signaling was associated to a total loss of NG2 chondroitin sulfate proteoglycan staining around the vessels (Supplementary Figure S1C, panel A). In contrast, the mPGES-1^−/−^ tumor vessels were positive for either NG2 (Supplementary Figure S1C, panel B), or α smooth muscle actin (αSMA) expression (Figure 2C), indicative of functional vessels [15]. In line with the *in vitro* data, VEGF and HIF-1α were over-expressed in mPGES-1^+/+^ compared to mPGES-1^−/−^ tumors (Figure 2D and Supplementary Figure S1D, panel a for HIF-1α). Together these results indicate that mPGES-1 expression is functionally associated with VEGF expression and tumor vascularization in DU145 cells.

### mPGES-1/PGE-2 signaling controls miRNA expression affecting its maturation

MiRNAs regulate angiogenesis [16, 17]. We investigated whether miRNA expression was involved in VEGF expression induced by mPGES-1/PGE-2 signaling in prostate cancer cells. DU145 mPGES-1^+/+^ and mPGES-1^−/−^ cells underwent miRNA expression-profiling using miRNA arrays containing 88 human miRNA probes (Supplementary Figure S2A).

We identified a total of 32 miRNAs, 30 of which were significantly down-regulated whereas two were up-regulated in mPGES-1^+/+^ relative to mPGES-1^−/−^ (> 2.5-fold change) (Supplementary Table S1).

**Figure 3:**
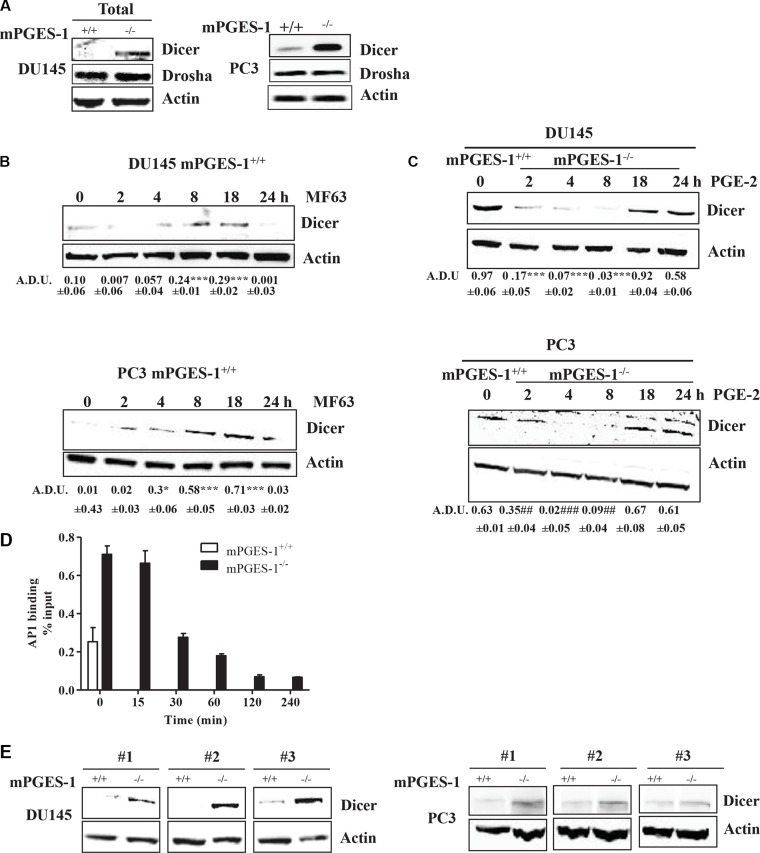
PGE-2 modulates Dicer transcriptional activity (**A**) Dicer and Drosha protein expression in total extract from DU145 and PC3 mPGES-1^+/+^ and from mPGES-1^−/−^ cells maintained for 18 h in 10% FBS. b-actin was used to normalize loading. *N* = 3. (**B**) Dicer protein expression in DU145 and PC3 mPGES-1^+/+^ exposed to MF63 (10 μM). (**C**) Dicer protein expression in DU145 and PC3 mPGES-1^−/−^ exposed to PGE-2 (1 μM). b-actin was used to normalize loading. *N* = 3. Data expressed in A.D.U. ***P < 0.001, *P < 0.05 vs. untreated cells. (**D**) Analysis of specific binding of AP1 to Dicer promoter region in mPGES-1^+/+^ and mPGES-1^−/−^ cells by EpiTect Chip qPCR primer assay. AP1 transcription factor was immunoprecipitated from cells in basal condition (10% FBS) or from mPGES-1^−/−^ stimulated with PGE-2 (1 μM). Immunoprecipitated DNA was amplified with specific primers for the Dicer proximal promoter region by QPCR. *N* = 3. Data is expressed as % of input. The % of input of the positive control (chromatin fragments isolated before immunoprecipitation) was 0.83 ± 0.02 SD. The % of input of the negative control, obtained by immunoprecipitation with normal rabbit serum, was 0.1 ± 0.0 SD. (**E**) Dicer protein expression in tumors from DU145 and PC3 mPGES-1^+/+^ or mPGES-1^−/−^ mice. b-actin was used to normalize loading. *N* = 3.

Since we postulated that PGE-2 might influence the miRNA processing by modulating Drosha or Dicer expression, we assessed protein expression of both enzymes in tumor cells. In total extract, Drosha protein was similar in both cell lines (Figure 3A), its localization being confined to the cell nucleus (Supplementary Figure S2B, middle lane, DU145 cells). Dicer levels were significantly lower in both total (Figure 3A) and cytoplasmic extract of mPGES-1^+/+^ cells (Supplementary Figure S2B, DU145 cells) than in mPGES-1^−/−^ cells. Dicer expression was undetectable in nuclear extract (Supplementary Figure S2B, DU145 cells). Consistently, MF63 (10 μM) significantly increased Dicer expression in mPGES-1^+/+^ cells (Figure 3B), whereas exogenous PGE-2 (1 μM, 2–24 h) decreased in mPGES-1^−/−^ cells (Figure 3C).

As the Dicer promoter has several binding sites for AP1 (cJUN+cFOS) and cJUN, we then determined the expression of cJUN and cFOS in both tumor cells to obtain insights into the mechanism whereby mPGES-1/PGE-2 signaling affects Dicer. cJUN and cFOS expression was found in the nuclear extract of DU145 mPGES-1^−/−^ cells, but was barely detectable in mPGES-1^+/+^ cells (Supplementary Figure S2C). Involvement of cJUN and cFOS in inducing Dicer expression was further documented by silencing them in mPGES-1^−/−^ cells. Knock down of cJUN and/or cFOS was sufficient to decrease expression of Dicer in mPGES-1^−/−^ (Supplementary Figure S3A). Additional evidence of a role of cJUN was obtained by chromatin immunoprecipitation assay. In basal conditions (10% FBS), cJUN binding to Dicer promoter was greater in mPGES-1^−/−^ than in mPGES-1^+/+^ cells (Figure 3D). Exogenous PGE-2 of mPGES-1^−/−^ cells decreased cJUN binding to Dicer promoter (Figure 3D).

Since cJUN and cFOS also regulate VEGF, we evaluated whether their inhibition in mPGES-1^−/−^ cells directly affected VEGF expression. cJUN or cFOS silencing was not sufficient to reduce VEGF expression (Supplementary Figure S3B). Finally, Dicer expression was up-regulated relative to mPGES-1^+/+^ in mouse mPGES-1^−/−^ xenografts *in vivo* (Figure 3E). These results suggest that mPGES-1/PGE-2 signaling down-regulates cJUN/AP1 and consequently Dicer and miRNA expression in prostate cancer cells.

### miR-15a and miR-186 controls VEGF expression *in vitro*

Among the miRNAs down-regulated by mPGES-1/PGE-2 signaling in DU145 cells, some have been involved in angiogenesis, others in inflammation or stemness (Supplementary Table S1). Five miRNAs (miR-15b, miR-93 miR-15a, miR-186 and miR-103) have been predicted to target VEGF and HIF-1α on the basis of DianaMT, PICTAR5, miRanda, miRBASE, miRWALK and Target Scan analysis (Supplementary Table S2). These five miRNAs were downregulated in mPGES-1^+/+^ cells (Supplementary Figure S2A). QPCR analysis provided further evidence of down-regulation of miR-15a, miR-186 and miR-103 in mPGES-1^+/+^ cells (Supplementary Figure S4A). miR-15b and miR-93 were not modified.

**Figure 4:**
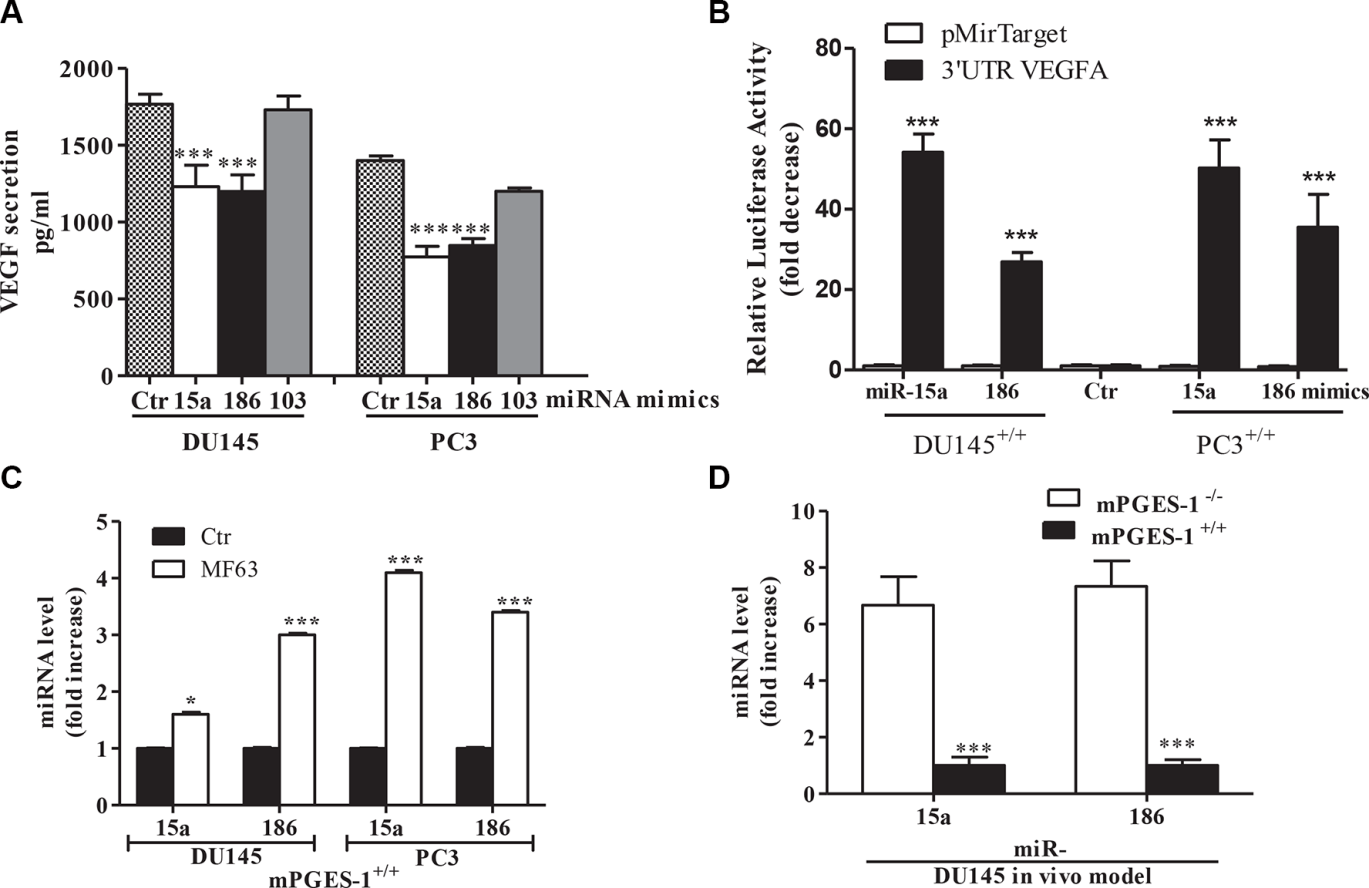
mPGES-1 down-regulates miR-15a and miR-186 upstream of VEGF expression (**A**) ELISA for VEGF in DU145 and PC3 mPGES-1^+/+^ cells (1% FBS, 48 h) transfected with miR-15a, miR-186 or miR-103 mimics (50 nM). ***P < 0.001 vs. mPGES-1^+/+^ control cells. (**B**) Endogenous requirement of miR-15a and miR-186 as VEGF inhibitors. Bars show expression of the VEGF 3′UTR reporters in DU145 and PC3 mPGES-1^+/+^ cells treated with miR-15a and miR-186 mimics. Data are reported as fold increase vs mPGES-1^+/+^ control cells. ***P < 0.001 vs. mPGES-1^+/+^ control cells. (**C**) miRNA levels measured by QPCR in DU145 and PC3 mPGES-1^+/+^ treated with MF63 (10 μM, 18 h). ***P < 0.001, *P < 0.05 vs. untreated cells (Ctr). (**D**) miRNA levels measured by QPCR in tumors from DU145 mPGES-1^+/+^ or mPGES-1^−/−^ mice. Data is reported as multiples of increase in expression in mPGES-1^+/+^ vs. mPGES-1^−/−^ cells (=1). *N* = 5. ***P < 0.001 vs. mPGES-1^−/−^ cells.

We also found that treatment of mPGES-1^+/+^ cells with miR-15a, miR-103 or miR-186 mimics (50 nM), besides increasing the endogenous pool of their respective miRNAs (Supplementary Figure S4B), down-regulated VEGF expression/production (*p* < 0.001, Supplementary Figure S4C, S4D, S4E and Figure 4A). The exception was miR-103, which failed to affect VEGF (Supplementary Figure S4C, S4D). Conversely, in mPGES-1^−/−^ cells, antagomirs for miR-15a and miR-186 (50 nM), which decreased the amount of detectable endogenous miR-15a or miR-186 (Supplementary Figure S5A), induced VEGF expression/production (Supplementary Figure S5B, S5C and S5D). Combination of miR-15a and miR-186 mimics or antagomirs did not show addictive effects.

To verify the putative direct interaction between miR-15a and miR-186 and the VEGF 3′-UTR, the 3′-UTR-luciferase reporter construct of VEGF and the control construct were independently transfected into the DU145 and PC3 mPGES-1^+/+^ cells. The activity of these reporters were evaluated in cells mPGEs-1^+/+^ treated with miR-15a, or miR-186 mimics.

The luciferase assays showed that compared with control, miR-15a and miR-186 dramatically decreased the luciferase activity of reporter plasmid with the VEGF 3′UTR. However, miR-15a and miR-186 had no effect on luciferase activity of pMir-target vector (Figure 4B). These data indicate that VEGF 3′UTR is a specific direct target of miR-15a and miR-186.

Consistently, PGE-2 treatment (1 μM) reversed miR-15a and miR-186 expression in mPGES-1^−/−^ cells (Supplementary Figure S6A and S6B, DU145 cells), corroborating the indication that mPGES-1/PGE-2 signaling is upstream of miRNAs. Moreover, MF63 increased levels of both miRs in mPGES-1^+/+^ cells (Figure 4C), and miR-15a and miR-186 were up-regulated with respect to mPGES-1^+/+^ tumors in DU145 mPGES-1^−/−^ xenografts *in vivo* (Figure 4D).

**Figure 5:**
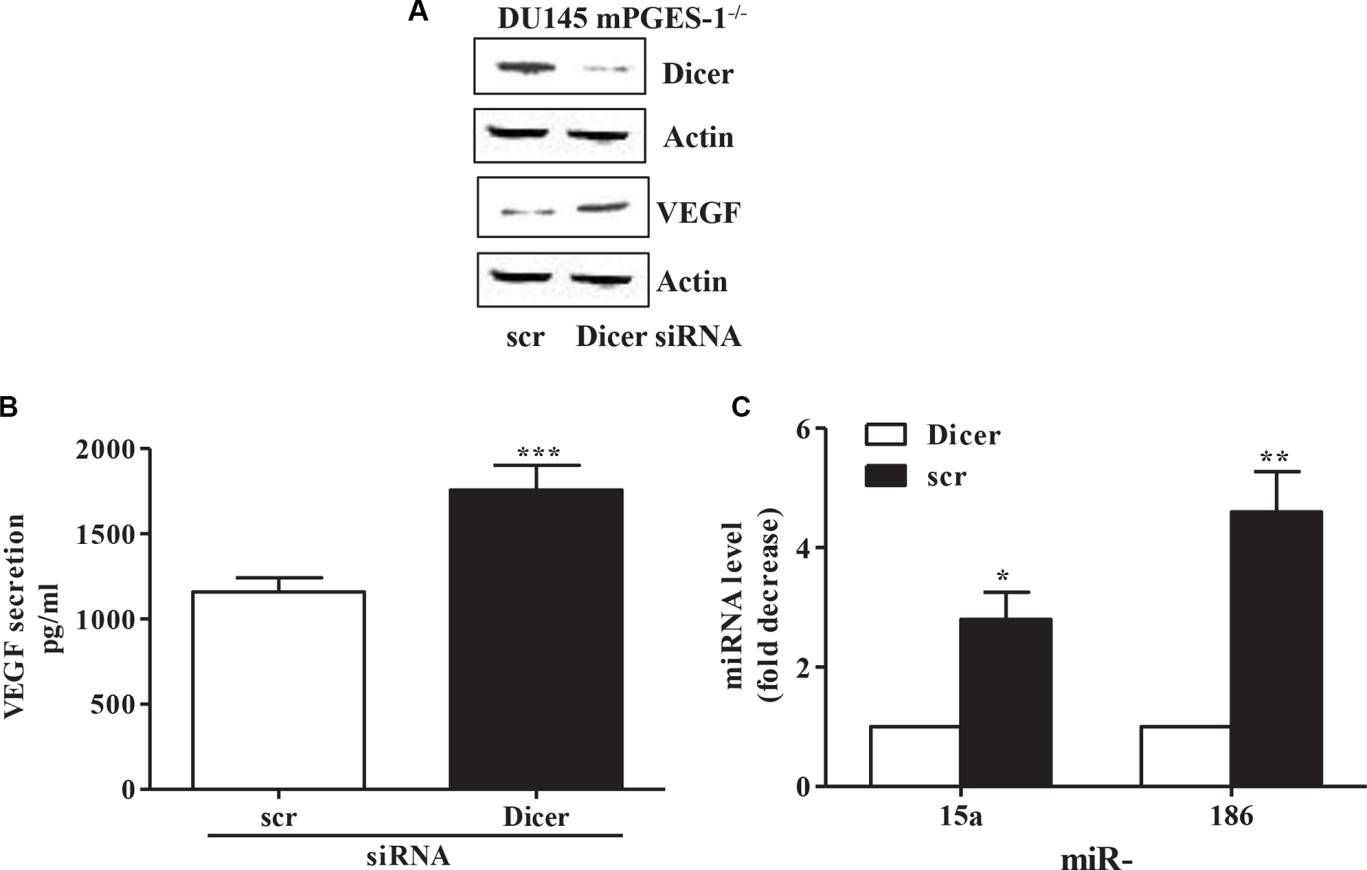
miRNA and VEGF expression/production in Dicer silenced mPGES-1^−/−^ cells (**A**) Dicer and VEGF protein expression in scrambled and in Dicer siRNA-transfected DU145 mPGES-1^−/−^ cells. b-actin was used to normalize loading. *N* = 3. (**B**) ELISA immunoassay for VEGF in scrambled and in Dicer siRNA-transfected DU145 mPGES-1^−/−^ cells. ***P < 0.001 compared to scrambled cells. (**C**) mRNA levels of mature miRNA measured by QPCR in DU145 mPGES-1^−/−^ cells silenced for Dicer. Results are expressed as multiples of increase in miRNA expression in DU145 mPGES-1^−/−^ scrambled vs. Dicer siRNA–transfected cells (=1). **p < 0.01, *p < 0.05 compared to Dicer siRNA-transfected mPGES-1^−/−^ cells.

Finally, silencing Dicer in DU145 mPGES-1^−/−^ cells promoted VEGF expression/secretion (Figure 5A and 5B) and down-regulated miR-15a and miR-186 (Figure 5C), indicating that mPGES-1/PGE-2 signaling decreases the miR-15a and miR-186/VEGF pathways through inhibition of Dicer expression.

### miR-186 controls tumor growth and VEGF output *in vivo*

**Figure 6:**
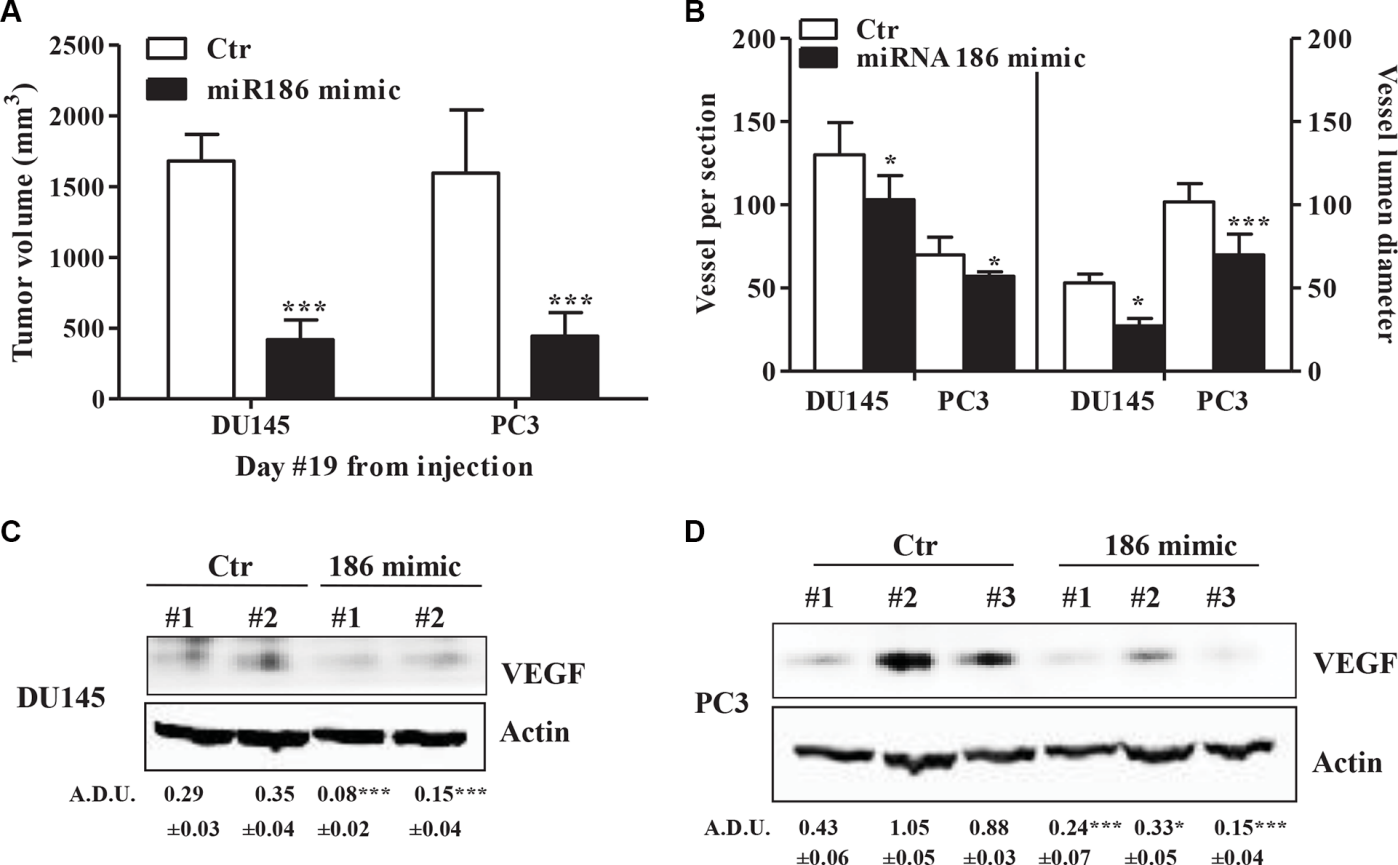
miR-186 controls tumor growth and angiogenesis *in vivo* (**A**) Antitumor activity evaluated in nude mice inoculated with DU145 and PC3 mPGES-1^+/+^ and treated subcutaneously with miR186 mimic or negative control. Data is expressed as tumor volume (mm^3^). ***P < 0.001 compared to mPGES-1^+/+^ treated with negative non-targeting control. Six nude mice per experimental group. (**B**) Vessel number and size in tumors from DU145 or PC3 mPGES-1^+/+^ treated with control or 186 mimic. Quantification of human CD31 and vessel lumen was performed by counting ten random fields/sections per slide; each slide had five sections. Data represents the number of vessels counted per section (left) or vessel diameter (μm) in tumors (right). ***p < 0.001, *p < 0.05 vs. control tumors. (**C**) VEGF protein expression in tumors from DU145 or (**D**) PC3 mPGES-1^+/+^ treated with control or 186 mimic. β-actin was used to normalize loading. *N* = 3. ***P < 0.001 vs. Ctr tumors.

The data presented so far underscores the potential link between miR-186 and VEGF/angiogenesis *in vivo*. To test this link, we assayed whether miR-186 inhibiting VEGF expression, could reduce angiogenesis and tumor size *in vivo*. miR-186 mimic treatment (3 μg/mouse) of DU145 and PC3 mPGES-1^+/+^ mouse xenograft led to a significant reduction in mPGES-1^+/+^ tumor size compared to control tumors (Figure 6A), showed lower vessel density and smaller luminal size (Figure 6B left and right graph, respectively), as well as a comparable reduction in VEGF protein expression (Figure 6C and 6D). This indicates that miR-186 is downstream of mPGES-1/PGE-2 and that it inhibiting VEGF in prostate cancer might decrease prostate cancer growth and angiogenesis.

### Tumor associated-mPGES-1/PGE-2 signaling promotes endothelial activation through VEGF release

Next we investigated the effect of mPGES-1/PGE-2 signaling on the activation of endothelial cells (a requirement during the angiogenesis process) *in vitro* using a co-culture system with endothelial cells and prostate cancer cells. When HUVEC were cultured with mPGES-1^+/+^ tumor cells, plating them in a thin Matrigel layer, they organized in a network of cord-like structures that invaded the gel (Supplementary Figure S7A, panel A). Conversely, when co-cultured with mPGES-1^−/−^ cells, the capacity of HUVEC to promote a cord-like network was lost (Supplementary Figure S7A, panel B vs. A), but restored by pre-treatment with PGE-2 (Supplementary Figure S7A, panel C vs. panel b, panel e for quantification). Evidence of the link between VEGF and mPGES-1 signaling was corroborated by experiments in which the VEGF neutralizing antibody (bevacizumab) added to DU145 mPGES-1^+/+^-HUVEC co-culture (Supplementary Figure S7A panel D) markedly reduced cord-like network formation (Supplementary Figure S7A panel D vs. panel C, panel e for quantification). These results indicate that mPGES-1/PGE-2 signaling induced robust activation of endothelial cells controlling the output of angiogenic factors in prostate cancer cells.

In line with the above results we found that treatment of DU145 mPGES-1^+/+^ cells with miR-15a or miR-186 mimics (50 nM) reduced the ability of HUVEC to form cord-like structures (Supplementary Figure S7B, panel B, C, and D). Conversely, antagomirs for miR-15a and miR-186 (50 nM) induced abundant sprouting in mPGES-1^−/−^ cells (Supplementary Figure S7C panel B, C, and D). Finally, silencing of Dicer in DU145 mPGES-1^+/+^ cells increased HUVEC-mediated sprouting in the co-cultured model (Supplementary Figure S7D, panel B, and C), indicating that mPGES-1/PGE-2 promotes activation of endothelial cells in prostate cancer cells by reducing Dicer, miR-15a and miR-186 expression, thus promoting VEGF secretion.

### Expression of mPGES-1 in prostate tumors associates with elevated VEGF expression and microvessel density

**Figure 7:**
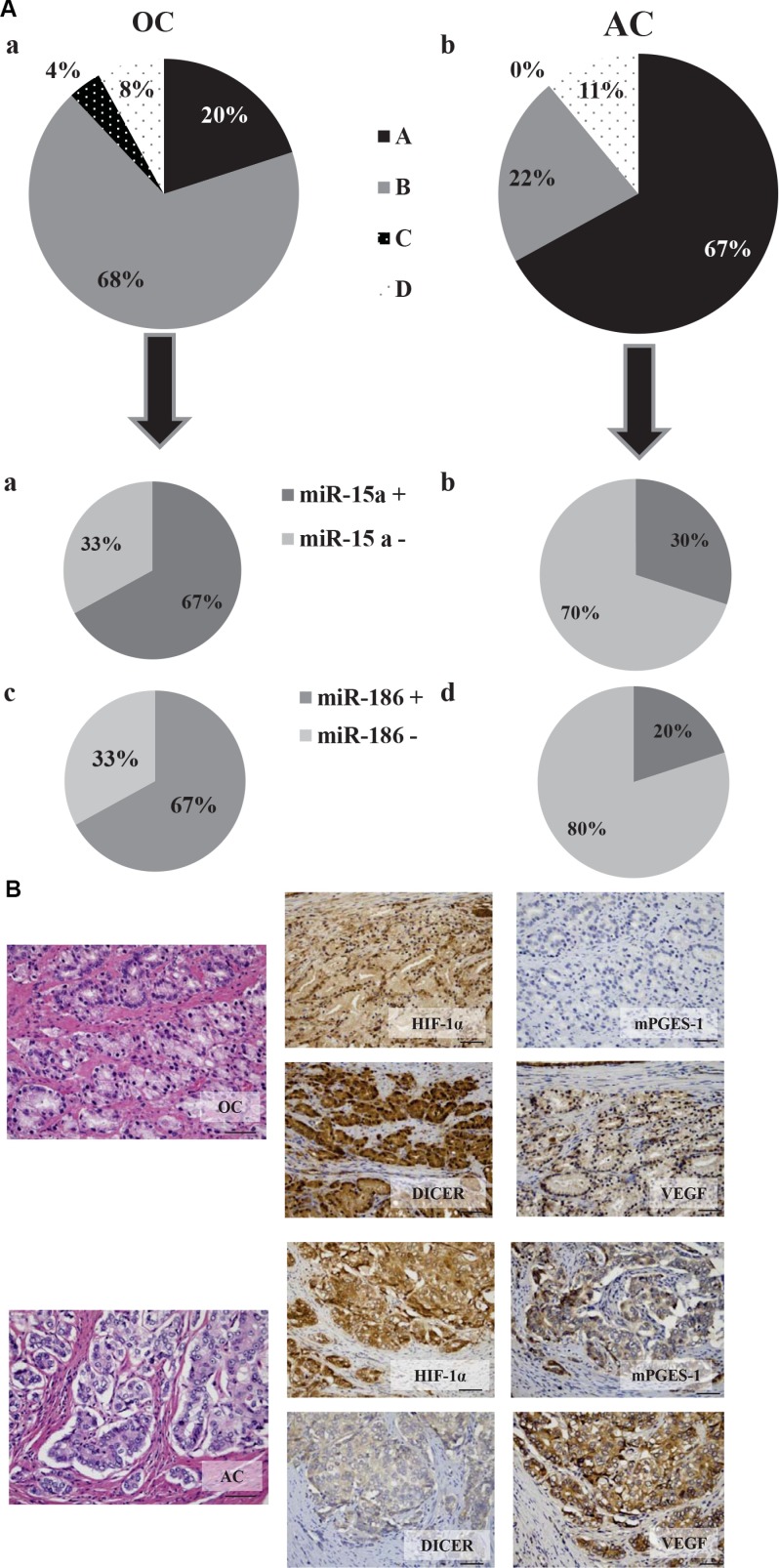

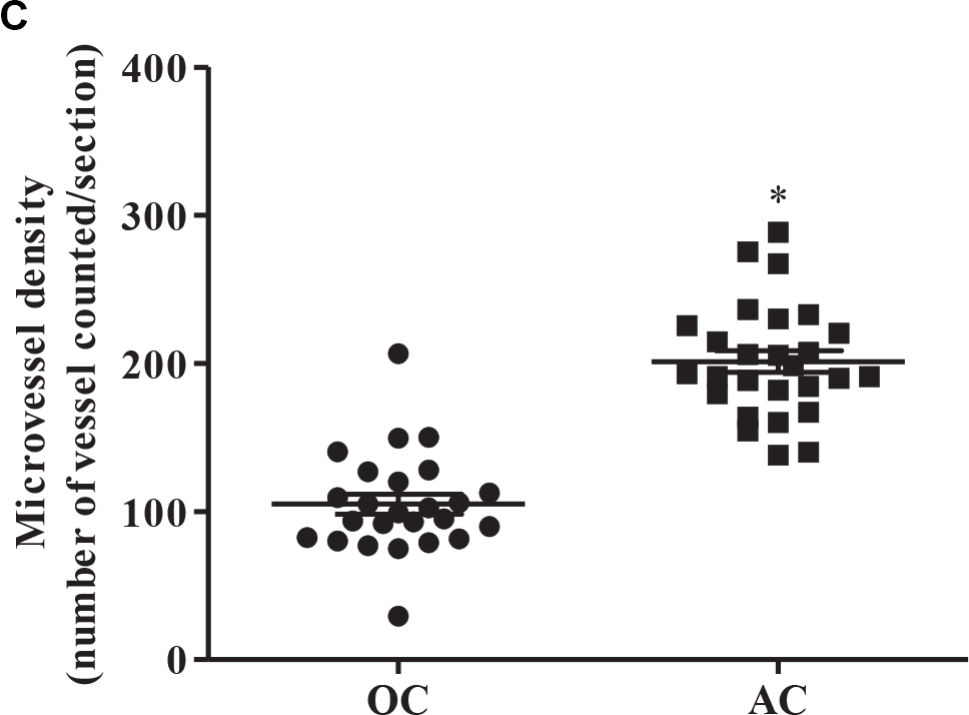
mPGES-1 expression in human prostate cancers is associated with elevated CD31, VEGF, and HIF-1α expression and reduced Dicer, miR-15a and −186 expression (**A**) Pie charts represent the percentage of expression of mPGES-1, VEGF, HIF-1α and Dicer in donors of prostate tissues (OC A and AC B). Black slice represents samples positive for mPGES-1, VEGF and HIF-1α and negative for Dicer; grey slice represents samples negative for mPGES-1, VEGF and HIF-1α and positive for Dicer; black slice with white dot indicates samples negative for mPGES-1, VEGF, HIF-1α and Dicer; squared slice shows samples positive for mPGES-1, VEGF, HIF-1α and Dicer (positive samples are calculated as sum of H-score 1, 2 and 3). (D) Pie charts display tumor grade and miR-15a (A and B) and miR-186 (**C** and **D**) expression in cancer samples. (**B**) Immunohistochemical analysis of mPGES-1, VEGF, HIF-1α and Dicer in organ-confined prostate cancers (OC) with low Gleason score (top lane) and in locally advanced prostate cancers (AC) with high Gleason score (bottom lane). The hematoxylin-eosin stained sections are included as basal control. Scale bars indicate 50 μm, magnification 25X. (C) Quantification of human CD31 was performed counting ten random fields/sections per slide; each slide had three sections. *P < 0.05; T3 prostate cancer (AC) compared to T2 prostate cancer (OC).

Prostate cancer tissue samples express mPGES-1 [5]. In order to investigate whether there was an association between mPGES-1, Dicer expression and VEGF/angiogenesis in these samples we assessed expression levels of mPGES-1 with Dicer and angiogenic markers (CD31, VEGF and HIF-1α) in human specimens with different tumor grade/staging. On histopathologic stratification of the tumors, we observed that mPGES-1 expression was clearly detectable in 21 of the 27 advanced cancers (AC) (78%, Supplementary Table S3), but in only 7 of the 25 organ-confined (OC) samples (28%, Supplementary Table S3). Consistently, in 21 out of 27 AC samples (78%), VEGF and HIF-1α also appeared to be high expressed (Figure 7A and 7B, bottom lane for representative images). In the AC samples, low expression of Dicer appeared to be associated with high expression of mPGES-1, VEGF and HIF-1α (66.7%, Figure 7A and 7B, bottom lane for representative images). Conversely, 72% of OC samples showed low expression of mPGES-1, VEGF and HIF-1α that was associated with high expression of Dicer Figure 7A and 7B, top lane for representative images). Moreover, when compared to OC samples, AC samples showed higher microvessel density as indicated by CD31 staining (Figure 7C, *p* < 0.01). In 6 OC and 10 AC samples we also investigated miR-15a and miR-186 expression. We observed that 66.7% of OC samples expressed significant levels of miR-15a and miR-186, whereas only 30% and 20% of AC samples expressed significant levels of miR-15a and miR-186, respectively (Figure 7A). A weak but negative association among miR-15a or miR-186 and mPGES-1 and VEGF expression in OC and AC tissue was noted. These miRNAs therefore appear to be elevated in samples with lower levels of mPGES-1 and VEGF. Collectively, these results suggest miR-15a and miR-186 as potential prognostic biomarkers in advanced prostate cancer linking high mPGES-1 levels with enhanced VEGF/angiogenic features.

## DISCUSSION

These results demonstrate that prostate cancer cells constitutively expressing mPGES-1, DU145 cells and PC3 cells, activate endothelial cells through output of VEGF, which was causally linked to PGE-2 production. Although the angiogenesis-promoting effect of PGE-2 in tumors has been described in a number of reports [2, 18–20], its mechanism remains to be elucidated. Here we showed that PGE-2 alters tumor miRNA biogenesis, resulting in increased VEGF expression and in tumor angiogenesis. These observations were gathered in prostate cancer cells, in one set of which the mPGES-1 enzyme was persistently knocked-down and/or deleted (mPGES-1^−/−^), or pharmacologically inhibited by the specific inhibitor MF63, while in a second set, cells were transfected with a control shRNA or CRISP/Cas9 plasmid (mPGES-1^+/+^). Evidence of miRNA involvement in mPGES-1/PGE-2 signaling-promoted tumor angiogenesis was gleaned over several experiments. For example: a) sharp downregulation of Dicer, cFOS and cJUN expression in mPGES-1^+/+^ cells with respect to mPGES-1^−/−^, b) decreasing angiogenic phenotype in mPGES-1^+/+^ cells after Dicer silencing, or silencing of its promoters (cFOS and cJUN); c) reduced binding of cJUN to Dicer in mPGES-1^+/+^, detected by chromatin immunoprecipitation; d) increased Dicer expression in mPGES-1^+/+^ after enzyme blockade by MF63.

Insights into the regulatory mechanism of miRNA were provided by gene profiling, which revealed that 32 out of 88 miRNA genes were significantly different (change by factor/quotient > 2.5) in mPGES-1^+/+^ cells, thirty and two of which were down- and up-regulated, respectively (see green vs. red color in the Supplementary Figure S2). Further analysis (QPCR) pointed to three miRNAs (miR-15a, −103, and −186). The functional relevance of the selected miRNA was demonstrated in cultured mPGES-1^+/+^ cells, where exposure to specific miRNA synthetic mimics reduced VEGF and impaired ability to elicit endothelial cell sprouting. The opposite emerged from experiments on mPGES-1^−/−^ cells incubated with synthetic antagomirs, where we observed a large increase in VEGF output and a rich network of cord-like structures of endothelial cells, similar to that obtained after exposure to PGE-2. PGE-2 showed a surprising ability to reverse the up-regulation of miR-15a and miR-186 in mPGES-1^−/−^ cells, which indicates that the effect occurred up-stream of the miRNA system. MiR-186 decreased in response to PGE-2 in a time dependent manner, while miR-15a showed a more complex kinetic, which might be associated with the complexity of the system or the technical issue [21]. Collectively, this study provides clear evidence that the oncogenic drive of prostate cancer cells is closely regulated by the expression of miRNAs which act by restraining the output of angiocrine factors from tumor cells. mPGES-1 plays a pivotal role in miRNA expression, as it decreases Dicer expression.

However, controversies on the role of Dicer in tumor growth have emerged since in prostate adenocarcinoma, increase and decrease of Dicer expression have both been reported [22, 23]. Nonetheless, suppressing Dicer activity in prostate cancer cells has been shown to reduce growth, but to lead to a more invasive phenotype [24]. Another caveat concerns the observed magnitude of mPGES-1 effect in tumor xenografts on VEGF/HIF-1α up-regulation and tumor growth, suggesting that other miRNAs than miR-15a and −186, downstream to Dicer inhibition, and other target than VEGF, might be involved [25–28]. In addition, as previously described for miR-15a, deregulated miRNAs might also affect stroma cell functions supporting tumor progression and angiogenesis [29, 30]. Reduced expression of miR-15a has been reported to be associated with anti-apoptotic, proliferative, invasive and angiogenic properties of cancer cells [31–33]. Other experiments comparing mPGES-1^+/+^ with mPGES-1^−/−^ cells helped to define the aggressive traits imparted to tumors by mPGES-1. In fact, when mPGES-1^+/+^ were co-incubated with HUVEC (Matrigel assay) we observed enhanced output of VEGF and abundant cord-like sprouting that specifically regressed with bevacizumab, a VEGF-neutralizing antibody. Moreover, when these cells were implanted in nude mice we detected enhanced VEGF and HIF-1α expression, accompanied by significantly enhanced tumor growth and vessel density compared to mPGES-1^−/−^ cells. An important experiment performed on mPGES-1^+/+^ mouse xenograft models clearly showed that sub-chronic treatment with miR-186 mimic significantly reduced tumor growth, angiogenesis and VEGF expression [34, 35, 36]. Thus, it appears that tumor-intrinsic mPGES-1 promotes VEGF expression and new vessel formation. an harbinger of dissemination favoring tumor expansion [19]. The involvement of mPGES-1 in prostate cancer progression is in line with observations in other tumors and underscores the role of an inflammatory milieu in the development of malignancies [5, 18, 20, 37–39]. Other evidence documents the role of miR-15a and miR-186 as pro-oncogenic molecules [25, 26], as clinical studies have observed that reduced expression of these miRNAs is associated with poor clinical prognosis in prostate cancer, and other tumors [25, 27, 28].

The translational relevance of these results is indicated by observations on human prostate cancer specimens from a small set of patients undergoing radical prostatectomy. The patients were assigned to organ-confined and advanced prostate cancer groups on the basis of PSA level, tumor stage and Gleason score. Given the small number of samples no statistical analysis was performed. Nonetheless, we recorded higher expression of mPGES-1, VEGF and HIF-1α together with lower Dicer expression in the advanced group (AC) contrasting with low expression of all parameters in the organ-confined (OC) group. Sharper differences in vessel density were also recorded, with the advanced group showing nearly three times the vessel density of the OC group. Moreover, high levels of miR-15a and −186 were more frequently expressed in organ-confined than in advanced tumor samples, demonstrating a direct association between the two miRNAs and VEGF expression.

In conclusion, the results demonstrate that high PGE-2 levels reduce Dicer expression and consequently miRNA biogenesis in prostate cancer cells. PGE-2-mediated downregulation of miR-15a and miR-186 is specifically related to VEGF production and angiogenesis. Considering the influence of miR-15a and miR-186 on angiogenesis and VEGF output in prostate cancer cells, we suggest that these miRs could be potential candidates for attenuating the aggressive traits of prostate cancer. This alternative approach might overcome the chemo-resistance often encountered with drugs targeting VEGF and/or VEGF receptors.

## MATERIALS AND METHODS

### Cell culture

DU145 wild type (WT, passages 5–20, ATCC^®^ HTB-81^™^, certified by STRA, LGC Standards S.r.l., Sesto San Giovanni, Milan, Italy) is a prostate cancer cell line with high constitutive expression of mPGES-1 [5]. DU145 mPGES-1 knockdown (mPGES-1^−/−^, passages 8–20) and control cells, transfected with scrambled non-target shRNA (mPGES-1^+/+^, passages 8–20) cells were obtained and cultured as described [5].

Three different mPGES-1-knockdown clones have been used Lenti vector plasmids for mPGES-1 and control were obtained from Sigma Aldrich (Supplementary Figure S8A). All the plasmids were sequence verified. The sequence of plasmid inserted in DU145 cells clone 1 is: 5′-CCGGGCTGCTGGTCATCAAGATGTACTCGAGTA CATCTTGATGACCAGCAGCTTTTTG-3′, the sequence of plasmid inserted in DU145 cells clone 2 is: 5′-CCGGGCTCTGCAGATCCTCTGGGAACTCGAGTT CCCAGAGGATCTGCAGAGCTTTTTG-3′. To generate mPGES-1 knockdown (−/−) cells, 1 × 106 HEK293 cells (Life Technologies) were transfected with 2.25 μg of PAX2 packaging plasmid (Addgene, Camb ridge, MA, USA), 0.75 μg of PMD2G envelope plasmid (Addgene), and 3 μg of pLKO.1 (Addgene) hairpin vector utilizing 12 μl of Lipofectamine 2000 on 10 cm plates. Polyclonal populations of transduced cells were generated by infection with 1 MOI (multiplicity of infectious units) of lentiviral particles. At 3 days post infection, cells were selected with 20 μg/ml neomycin/kanamycin (Sigma Aldrich) for 1 week.

Compared to DU145 wild type (WT), transfection of cells with the scrambled non-target shRNA (mPGES-1^+/+,^ detail in Supplementary data) did not affect mPGES-1/PGE-2 and VEGF expression/production (Supplementary Figure S8B–S8D). Further, differences in the mPGES-1/PGE-2 expression/secretion did not affect the *in vitro* proliferation rate of these two cell lines (Abs 0.98 ± 0.07 and 0.91 ± 0.1 for DU145 mPGES-1^+/+^ and mPGES-1^−/−^ cells, respectively, measured by MTT assay).

PC3 wild type (WT, passages 8-20, ATCC^®^ CRL-1435^™^, certified by STRA) prostate cancer cells were from ATCC, Milan, Italy. Cells were grown in RPMI 1640 (Sigma Aldrich, St. Louis, MO, USA) supplemented with 10% FBS. Gene silencing of mPGES-1 in PC3 cells by CRISPR/Cas9 technology is reported below (Supplementary Figure S8E). In this cell model, mPGES-1 expression slightly increases the *in vitro* proliferation rate: (0.93 ± 0.08 and 0.65 ± 0.3 for PC3 mPGES-1^+/+^ and mPGES-1^−/−^ cells, respectively, measured by MTT assay). Compared to PC3 wild type (WT), transfection of cells with control CRISPR/Cas9 vector (mPGES-1^−+/+^) did not affect mPGES-1/PGE-2 and VEGF expression/production or *in vitro* proliferation rate (Supplementary Figure S8F–S8H; Abs 0.77 ± 0.03 and 0.82 ± 0.08 for PC-3 mPGES-1^+/+^ and mPGES-1^−/−^ cells, respectively).

DU145 and PC3 WT prostate cancer cell lines were immediately expanded after delivery (up to 6 × 10^7^ cells) and frozen down (1 × 10^6^/vial) such that both cell lines could be restarted after a maximum of 10 passages every 3 months from a frozen vial of the same batch of cells. Control of mycoplasma was done from a frozen vial.

Human umbilical vein endothelial cells (HUVEC, passages 3–10) were from Lonza, Milan, Italy (C2519A, certified for expression of CD31/105, von WFVIII, and positivity for acetylated low density lipoprotein uptake). Cells were grown in endothelial growth medium (EGM-2) (Clonetics, Milan, Italy) supplemented with 10% FBS. All cells remained in culture for less than 6 months.

### Knockout of PTGES1 gene by CRISPR/Cas9-mediated genome editing

PC3 cells were transfected with 1.5 μg RNA CRISPR plasmids (Santa Cruz, Santa Cruz, CA, USA) using Lipofectamine 2000 (Life Technologies, Carlsbad, CA, USA) according to the manufacturer's instructions. Beginning the day after transfection, these cells were treated with 1 μg/ml of puromycin. Surviving cells were reseeded at 1 cell per well in a 96-well plate. Expression of mPGES-1 in expanded colonies was detected by immunoblotting to select the mPGES-1-depleted colonies. Three different mPGES-1-depleted clones have been used.

### Reagents

Reagents were as follows: PGE-2 (Sigma Aldrich); Lipofectamine 2000 (Life technologies); MF63 (AbMole Biosciences, Colleretto Giacosa, TO, Italy); DY554 phalloidin (Thermo Fisher Scientific, Waltham, MA, USA), puromycin (Gibco, Thermo Fisher Scientific).

### Immunoblot analysis

Total protein lysates were obtained as previously described [18]. The following antibodies were used: VEGF (Merck Millipore, Darmstadt, Germania), Dicer (Abcam, Cambridge), Drosha (Cell Signalling, Danvers, MA, USA) all 1:1000, mPGES-1 (1:200, Cayman chemicals, Ann Arbor, Mi, USA) or HIF-1α (1:300, BD-Transduction Laboratories, Milan, Italy).

### Nuclear/cytoplasm translocation

To assess translocation of Dicer, Drosha, cJUN and cFOS from cytosol to nucleus, 8 × 10^5^ cells were plated in 10 cm diameter dishes, maintained in 10% FBS for 18 h and then scraped, homogenized on ice in a lysis buffer containing (in mM) 10 HEPES, 1 DTT, 10 KCl, 50 NaF, 0.1 EDTA, 0.1 EGTA, 1 Na_3_VO_4_, 0.5 PMSF and 0.1 NP-40 at 4°C, and centrifuged at 1000 *g* for 10 min to separate the nuclei. The supernatant was centrifuged at 13,200 *g* for 5 min to yield the cytosolic fraction. The nuclear fraction was lysed in buffer containing (in mM) 20 HEPES, 1 EDTA, 1 EGTA and 0.5 PMSF and analysed for cFOS (Merck Millipore), Dicer (Abcam), Drosha and cJUN (Cell Signaling), all 1:1000. Western blot was performed as described [18]. Images were digitized with the program CHEMI DOC Quantity One, blots were analyzed in triplicate by densitometry using NIH Image 1.60B5 software, and the results in arbitrary densitometric units (A.D.U.) were normalized for β-actin, β-tubulin or lamin (Sigma Aldrich).

### VEGF immuno-assays

VEGF was determined in supernatant using a Quantikine kit (R&D System, Milan, Italy). 3 × 10^4^ cells were exposed to 10% FBS or to PGE-2 (1 μM) for 48 h or siRNA-transfected for Dicer or transfected with mimics for miR-15a, miR-186, miR-103 or with miRNA inhibitors for miR-15a and miR-186. The conditioned media were collected, diluted in the standard diluents, and assayed as indicated in manufacturer's instructions.

### QPCR

Total RNA was obtained using an RNA mini kit (Qiagen, Inc., Milan, Italy). RNA (0.5 μg) was reverse transcribed using a RT-PCR kit (Applied Biosystems, Foster City, USA). Used as an internal control, GAPDH was assessed using premixed reagents from Applied Biosystems. VEGF mRNA detection was measured using the optimized TaqMan assay-on-demand (Applied Biosystems) and GAPDH detection was performed using TaqMan Universal PCR Master Mix (Applied Biosystems). The results were expressed as fold increase ^−ΔCt^. miRNA expression was measured by QPCR according to the manufacturer's instructions (miScript Primer Assay, Qiagen) and the results are expressed as multiples of increase or 2^^−ΔCt^. The mature miRNA sequences used are listed in Supplementary Table S2.

### Assay of luciferase activity

3′UTR of VEGF-A construct and pMir-Target Vector were obtained from Origene (Rockville, MD;USA) and used according to the manufacturer's instructions protocol Cells were harvested 48 h after co-transfection of miRNA mimics with reporter vector and assayed with Dual Luciferase Assay (Promega, Milan, Italy) according to the manufacturer's instructions protocol.

### *In vitro* endothelial cell sprouting

Tube formation assay was assessed as described [40]. For detail see supplementary data.

### siRNA/miRNA transfection

The siRNAs sequences: human cJUN (5′-AAGAACGTGACAGATGAGCAG-3′) and human cFOS (5′-AACCTGCTGAAGGAGAAGGAA-3′), were from Qiagen. The day before transfection, cells were trypsinized, and 2 × 10^5^ cells were seeded in 6-well plates. Transient transfection of siRNA was carried out using Lipofectamine 2000 (Life Technologies) according to the manufacturer's instructions. Cells were assayed 48 h after transfection.

The miRNA mimics and inhibitors for miR-103, miR-186 and miR-15a were from Qiagen and transfection was performed with Lipofectamine 2000 (Life Technologies) following the manufacturer's protocol. Oligonucleotides were used at a final concentration of 50 nM in antibiotic-free opti-modified Eagle's medium (Life Technologies). miRNA levels were validated by QPCR. Conditioned media and cell extracts were prepared for analysis 48 h after transfection.

Expression of 88 mature human miRNAs in DU145 cells was profiled using QPCR (miFinder, Sabiosciences, Qiagen). Gene expression data was normalized to SNORD 44, SNORD 47 and SNORD 48. RNU6-2, miRTC and PPC were controls for the array. Relative expression was determined for each of the 88 miRNAs using the formula 2^^−ΔCt^.

To assess differential miRNA expression in scrambled, Dicer siRNA-transfected DU145 mPGES-1^−/−^ cells, we isolated total RNA using miRNeasy Mini kit (Qiagen). miRNA expression was measured by QPCR according to the manufacturer's instructions (miScript Primer Assay, Qiagen). Gene expression data was normalized to SNORD 48. The mature miRNA sequences used are listed in Supplementary Table S2. miRNA mimics and inhibitors target the following mature miRNA sequences: for miR-186-5p (5′-CAAAGAAUUCUCCUUUUGGGCU-3′), for miR-15a-5p (5′-UAGCAGCACAUAAUGGUUUGUG-3′) and for miR-103-3p (5′-AGCAGCAUUGUACAGGGCU AUGA-3′).

### miRNA expression analysis

For determination of global baseline miRNA expression, total RNA was isolated from tumor cells using miRNeasy Mini kit (Qiagen).

### Chromatin immunoprecipitation assay

Chromatin immunoprecipitation assays were performed as previously described [41]. Experimental details are reported in supplementary data.

### Tumor xenograft

Experiments have been performed in accordance with the EEC guidelines for animal care and welfare (EEC Law No. 86/609) and National Ethical Committee. As recommended by EEC guidelines and Italian National laws for animal experimentation, to investigate the role of miR-15a and miR-186 mimics and inhibitors on VEGF expression and growth of DU145 and PC3 xenografts, we minimized the number of animals focusing on miR-186 mimic. The experiments were approved from Italian Health Ministry, d.m. n° (215/2011-B). Samples were obtained as previously described [18]. Details are in supplementary data.

### Human PCa specimen analysis

Radical prostatectomy specimens were collected from the University Hospital of Florence after written informed consent to perform this analysis was obtained from all patients. The hematoxylin-eosin stained sections were reviewed to confirm the diagnosis. Twenty-five carcinomas were limited to the prostate (OC, pT2) and were moderately differentiated (Gleason score = 6), whereas 27 cases were non-organ-confined (locally advanced PCa, AC, pT3/pT4) with a high Gleason score (≥ 7). Patients’ median age was 69 years (range 41–80 years).

Representative formalin-fixed, paraffin-embedded tumor tissue blocks were selected and 4-μm sections for each lesion were prepared for immunohistochemical analysis. Antigen retrieval was performed for 20 min in citrate buffer (pH 6.6) in a microwave at 500 W for VEGF, mPGES-1, HIF-1α and Dicer, and for 30 min in TRIS buffer (pH 9) in a microwave at 500 W for CD31. The sections were then allowed to cool down to room temperature for 20 minutes. After inactivating endogenous peroxidase activity and blocking cross-reactivity with 3% BSA, the slides were incubated at 4°C for 18 h with a dilute solution of mPGES-1 (1:50, Thermo Scientific), VEGF (1:100, Merck Millipore), CD31 (1:50, Dako, Cernusco sul Naviglio, MI, Italy), Dicer (1:100, Santa Cruz, Santa Cruz, CA, USA) and HIF-1α (1:100, Thermo Scientific). Location of primary antibodies was achieved by subsequent application of biotin-conjugated anti-primary antibody, streptavidin-peroxidase and diaminobenzidine (Sigma Aldrich). The staining was developed using a commercial immunoperoxidase staining kit following the manufacturer's instructions (biotin-streptavidin complex method, Merck Millipore). The slides were counterstained with hematoxylin.

Immunohistochemical staining was interpreted by two experienced pathologists without knowledge of the clinical data associated with each specimen. An H-score was calculated by using intensity (score of 3: strongly staining; score of 2: moderately staining; score of 1: weakly staining; score of 0: no staining) × percentage of tumor tissue stained (score of 1: 0–25%; score of 2: 26–50%, score of 3: 51–75%; score of 4: 76–100%) for each case.

### Purification of total RNA, including miRNA, from FFPE tissue sections

Formalin-fixed, paraffin-embedded sections (10 μm thick) of human prostate cancer were deparaffinized with deparaffinization solution (Qiagen) and purified with a miRNeasy FFPE kit (Qiagen) according to the manufacturer′s instructions.

### Immunohistological analysis

Immunohistological analysis was performed as previously described [18]. Experimental details of immunohistological staining of tumor tissues are reported in supplementary data.

### Statistical analysis

Results are expressed as means ± SD. Statistical analysis was carried out using using Student *t* test when appropriate and Bonferroni test (GraphPad) for multiple comparison. *P* < 0.05 was considered statistically significant.

## SUPPLEMENTARY MATERIAL FIGURES AND TABLES


