# Supplementary material for: Linking microsomal prostaglandin E Synthase-1/PGE-2 pathway with miR-15a and −186 expression: Novel mechanism of VEGF modulation in prostate cancer
Source: Oncotarget. 2016 Jun 14;7(28):44350–64. doi: 10.18632/oncotarget.10051 (PMC5190102; doi:10.18632/oncotarget.10051)
Supplement: Supplementary file 1 [file oncotarget-07-44350-s001.pdf]

Linking microsomal prostaglandin E Synthase-1/PGE-2 pathway with miR-15a and -186 expression: Novel mechanism of VEGF modulation in prostate cancer

Supplementary Materials

SUPPLEMENTARY METHODS

Cell culture

Human prostate cancer cells, DU145 mPGES-1 knockdown (mPGES-1$^{-/-}$) and non-target shRNA (mPGES-1$^{+/+}$) were cultured as described [1]. The DU145 WT cell line was transfected with shRNA plasmids using Lipofectamine 2000 (Life technologies, Carlsbad, CA). Stably transfected clones were isolated with puromycine (8 µg/mL). The sequence of the shRNA insert is: 5′-CCGGGAACGACATGGAGACCATCTACT CGAGTAGATGGTCTCCATGTCGTTCTTTTTG-3′. The underlined residues match nucleotides 236–258 in mPGES-1 mRNA.

Human umbilical vein endothelial cells (HUVEC) were purchased from Promocell (Heidelberg, Germany) and grown in endothelial growth medium (EGM-2) (EBM-2, containing 2% FBS, VEGF, R^3-IGF-1, hEGF, hFGF, hydrocortisone, ascorbic acid, heparin and GA-1000) (Clonetics, Cambrex Bio Science Walkersville, MD, USA), supplemented with 10% FBS. Cells were grown normally at 37ºC in 5% CO_2.

Endothelial cell sprouting assay

HUVEC cells (6 × 10^4 cells/well in EBM) were plated onto a thin layer (250 µL) of basement membrane matrix (Matrigel; BD Biosciences, Becton Dickinson, Waltham, MA, USA) in 24-well plates and co-cultured with WT or mPGES-1$^{+/+}$ tumor cells treated with 10% FBS medium, or mPGES-1$^{-/-}$ tumor cells with or without PGE-2 (1 µM) in 10% FBS, or DU145 mPGES-1$^{-/-}$ cells transfected with scramble or Dicer siRNA, or mPGES-1$^{-/-}$ cells transfected with miRNA inhibitors, or mPGES-1$^{+/+}$ cells transfected with miRNA mimics. Co-cultures were incubated at 37°C in 5% CO_2. After 12 h, the medium was removed and the cells were fixed and stained using DY554 phalloidin (Thermo Fisher Scientific, Waltham, MA, USA) for Factin. Images (magnification 4X) were obtained with a Nikon Eclipse TE 300 inverted microscope (4X/0.13). Quantification of cord-like structures and photomicrographs were performed as previously described [2].

In vivo tumor xenograft study

Experiments observed EEC guidelines (Law No. 86/609) and National Ethical Committee rules. DU145 (20^6) and PC3 (10^6) cells were injected s.c. into the flank of 5-week-old male athymic nude mice. Tumor size was determined by measuring tumor length and width and calculating tumor weight (mg) [tumor length × (tumor width)2]/2, where tumor length is the longest dimension (mm) and tumor width is the shortest dimension (mm). Where indicated, at 1 week after tumor cell implant, mice were injected subcutaneously with mimic miR186 (3 µg/mouse) or negative control miRNA with *in vivo* jetPEI as vehicle (Polyplus transfection, Bioparc, Illkirch, France). Treatment was repeated three times/week for a total of 8 injections. No side effects such as changes in mouse body weight, behavioural changes or other signs of discomfort were observed. 48 h after the last treatment, mice were sacrificed (by CO_2 asphyxiation 19 days after the first treatment and/or tumor volume measurement). Tumors were harvested and used to analyse protein [3], mRNA and miRNA expression or embedded in Tissue-Tek O.C.T. (Sakura, San Marcos, CA, USA) for histological examination. Tumor volume was expressed in mm3. Efficient delivery of miR-186 mimic was confirmed by qPCR (expression of mature miR-186 increased by a factor of ~4.3 in miR-186-treated with respect to control treated mice; $P < 0.001$). Cryostat sections (6 µm thick) of tissue samples were processed for immunohistochemical staining.

Immunohistochemistry

For histopathological analysis of CD31 we used hematoxylin and immunohistochemical staining. First, sections were fixed with acetone at –20°C for 5 min. After inactivating of endogenous peroxidase activity and blocking cross-reactivity with 3% BSA, the sections were incubated at 37°C for 1 h with dilute CD31 solution (1:100, Chemicon, Merck-Millipore, Darmstadt, Germania). Primary antibodies were located by subsequent application of biotin-conjugated antiprimary antibody, streptavidin-peroxidase and diaminobenzidine (Sigma Aldrich, Saint

Louis, MO, USA). The stain was developed using a commercial immunoperoxidase staining kit according to the manufacturer's instructions (biotin-streptavidin complex method, Merck-Millipore). The slides were counter-stained with hematoxylin. Negative controls were established by replacing the primary antibody with PBS. Specific staining for CD31 was scored as positive or negative according to the presence or absence of brown staining. Images were analyzed using a Nikon Eclypse T200 at magnification 20X/0.40.

Chromatin immunoprecipitation assay

Chromatin immunoprecipitation assay (ChIP) assays were performed as previously described [4]. In Brief DU145 cells (3×10^6) were maintained in RPMI with 10% FBS for 24 h and stimulated with PGE-2 for the indicated times. Cells were then fixed with 1% formaldehyde for 5 min at 37°C and lysed in ice-cold lysis buffer (10 mM HEPES, 1.5 mM $MgCl^2$, 10 mM KCl, 0.5 mM DTT, 0.1% NP-40 and protease inhibitors) for 10 min at 4°C. The nuclei pellet was suspended in nuclear lysis buffer (50 mM Tris–HCl pH 8, 10 mM EDTA, 1% SDS and protease inhibitors) and incubated on ice for 10 min. DNA was sheared by sonication and lysates were cleared by centrifuging and diluted in ChIP dilution buffer (50 mM Tris–HCl pH 8, EDTA 5 mM, NaCl 200 mM, and 0.5% NonidetP-40). Lysates were precleared with salmon sperm/protein A-agarose. A sample of "input DNA" was collected at this point. Protein–DNA complexes were immunoprecipitated overnight at 4°C with 2 μg of the corresponding antibody or non-immune rabbit serum as a control. Antibody–protein–DNA complexes were then captured using salmon sperm DNA/protein agarose for 30 min followed by washes with wash buffer (20 mM Tris–HCl pH 8, 2 mM EDTA, 0.1% SDS, 1% NP-40 and 500 mM NaCl) and TE buffer (20 mM Tris–HCl and 2 mM EDTA). The protein/DNA complexes were eluted using extraction buffer (20 mM Tris–HCl, 2 mM EDTA and 2% SDS) and disrupted by heating at 65°C overnight followed by proteinase K treatment for 2 h at 45°C. DNA was extracted with a Purification kit (Sigma Aldrich). QPCR was conducted using promoter specific primers from Qiagen (20 mM Tris–HCl pH 8, 2 mM EDTA, 0.1% SDS, 1% NP-40 and 500 mM NaCl) and TE buffer (20 mM Tris–HCl and 2 mM EDTA). The protein/DNA complexes were eluted using extraction buffer (20 mM Tris–HCl, 2 mM EDTA and 2% SDS) and disrupted by heating at 65°C overnight followed by proteinase K treatment for 2 h at 45°C. DNA was extracted with a Purification kit (Sigma Aldrich). QPCR was conducted using promoter specific primers from Qiagen (GPH1018139(+)05A, in position 4930).

Statistical analysis

Results are expressed as means ± SD. Statistical analysis was performed using ANOVA followed by Bonferroni test and Student t test when appropriate (GraphPad). $P<0.05$ was considered statistically significant.

REFERENCES

1. Hanaka H, Pawelzik SC, Johnsen JI, Rakonjac M, Terawaki K, Rasmuson A, Sveinbjörnsson B, Schumacher MC, Hamberg M, Samuelsson B, Jakobsson PJ, Kogner P, Rådmark O. Microsomal prostaglandin E synthase 1 determines tumor growth in vivo of prostate and lung cancer cells. Proc Natl Acad Sci USA. 2009; 106:18757–18762.
2. Finetti F, Terzuoli E, Bocci E, Coletta I, Polenzani L, Mangano G, Alisi MA, Cazzolla N, Giachetti A, Ziche M, Donnini S. Pharmacological inhibition of microsomal prostaglandin E synthase-1 suppresses epidermal growth factor receptor-mediated tumor growth and angiogenesis. PLoS One. 2012; 7:e40576.
3. Terzuoli E, Donnini S, Giachetti A, Iñiguez MA, Fresno M, Melillo G, Ziche M. Inhibition of hypoxia inducible factor-1alpha by dihydroxyphenylethanol, a product from olive oil, blocks microsomal prostaglandin-E synthase-1/vascular endothelial growth factor expression and reduces tumor angiogenesis. Clin Cancer Res. 2010; 16:4207–4216.
4. Donnini S, Finetti F, Terzuoli E, Giachetti A, Iñiguez MA, Hanaka H, Fresno M, Rådmark O, Ziche M. EGFR signaling upregulates expression of microsomal prostaglandin E synthase-1 in cancer cells leading to enhanced tumorigenicity. Oncogene. 2012; 31:3457–3466.

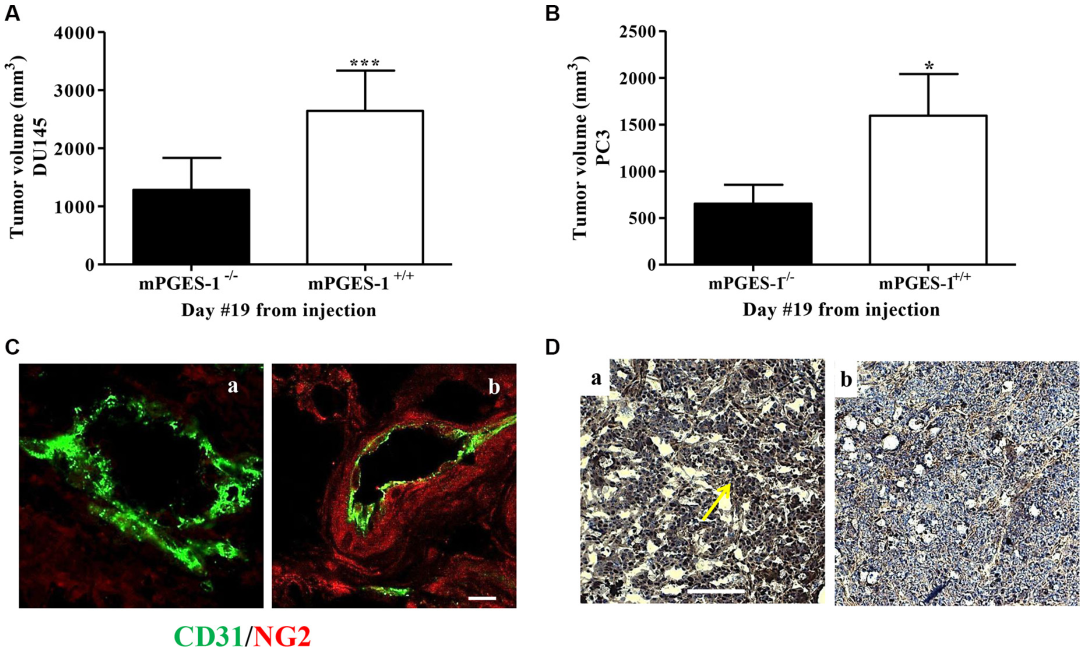

Supplementary Figure S1: Effect of mPGES-1 expression on tumor growth *in vivo.* Antitumor activity evaluated in nude mice inoculated with DU145 (**A**) or PC3 (**B**) mPGES-1$^{+/+}$ or mPGES-1$^{-/-}$ cells (19 days). Data is expressed as tumor volume (mm^3); ****P* < 0.001, **P* < 0.05 compared to mPGES-1$^{+/+}$ (7 nude mice per experimental group). (**C**) Representative images of double-immunostaining for CD31 (green) and NG2 (red) in tumor sections mPGES-1$^{+/+}$ (A) or mPGES-1$^{-/-}$ (A) mice. Scale bars indicate 80 µm. Images obtained with confocal microscopy at 60X magnification. (**D**) HIF-1α expression in tumor sections from mPGES-1+/+ (A) or mPGES-1-/- (B) mice. Scale bars indicate 50 µm. Images at 20X magnification.

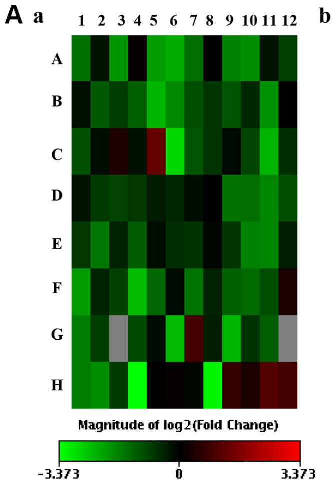

b

	1	2	3	4	5	6	7	8	9	10	11	12
A	hsa-miR-142-5p -2.77	hsa-miR-16 -1.18	hsa-miR-142-3p -3.94	hsa-miR-21 1.06	hsa-miR-15a -4.16	hsa-miR-29b -4.97	hsa-let-7a -2.73	hsa-miR-126 -1.02	hsa-miR-143 -3.32	hsa-let-7b -3.72	hsa-miR-27a -1.18	hsa-let-7f -1.80
B	hsa-miR-9 -1.13	hsa-miR-26a -2.23	hsa-miR-24 -1.74	hsa-miR-30e -2.41	hsa-miR-181a -5.31	hsa-miR-29a -3.55	hsa-miR-124 -2.00	hsa-miR-144 -1.63	hsa-miR-30d -2.18	hsa-miR-19b -1.45	hsa-miR-22 -3.79	hsa-miR-122 1.00
C	hsa-miR-150 -2.12	hsa-miR-32 -1.11	hsa-miR-155 1.32	hsa-miR-140-5p -1.16	hsa-miR-125b 2.68	hsa-miR-141 -7.31	hsa-miR-92a -2.18	hsa-miR-424 -1.60	hsa-miR-191 -1.09	hsa-miR-17 -1.91	hsa-miR-130a -5.23	hsa-miR-20a -1.51
D	hsa-miR-27b -1.20	hsa-miR-26b -1.71	hsa-miR-146a -1.85	hsa-miR-200c -1.67	hsa-miR-99a -1.26	hsa-miR-19a -1.45	hsa-miR-23a -1.12	hsa-miR-30a -1.00	hsa-let-7i -2.83	hsa-miR-93 -2.73	hsa-let-7c -3.42	hsa-miR-106b -2.06
E	hsa-miR-101 -1.68	hsa-let-7g -3.00	hsa-miR-425 -1.35	hsa-miR-15b -2.56	hsa-miR-28-5p -1.12	hsa-miR-18a -1.54	hsa-miR-25 -1.59	hsa-miR-23b -1.05	hsa-miR-302a -1.65	hsa-miR-186 -3.39	hsa-miR-29c -3.43	hsa-miR-7 -1.32
F	hsa-let-7d -4.18	hsa-miR-30c -1.40	hsa-miR-181b -1.84	hsa-miR-223 -5.65	hsa-miR-320a -2.55	hsa-miR-374a -1.09	hsa-let-7e -2.91	hsa-miR-151-5p -1.36	hsa-miR-374b -2.39	hsa-miR-196b -2.65	hsa-miR-140-3p -1.99	hsa-miR-100 1.29
G	hsa-miR-103 -3.14	hsa-miR-96 -1.77	hsa-miR-302b 1.06	hsa-miR-194 -1.93	hsa-miR-125a-5p -1.10	hsa-miR-423-5p -5.51	hsa-miR-376c 2,55	hsa-miR-195 -1.31	hsa-miR-222 -5.27	hsa-miR-28-3p -1.58	hsa-miR-128 -2.34	hsa-miR-302c 1.06
H	hsa-miR-423-3p -3.18	hsa-miR-185 -3.66	hsa-miR-30b -1.71	hsa-miR-210 -10.36	SNORD48 1.02	SNORD47 1.06	SNORD44 -1.08	RNU6-2 -9.50	miRTC 1.63	miRTC 1.29	PPC 2.10	PPC 1.82

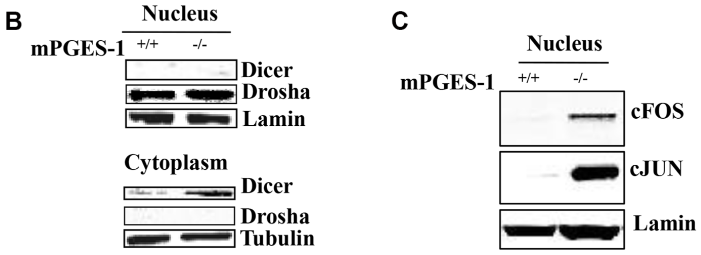

Supplementary Figure S2: Tumor-derived PGE-2 modulates miRNA levels in DU145 cells. (**A**) Heat map of up- (red), down- (green) regulated and unmodified (black) miRNAs in DU145 mPGES-1$^{+/+}$ vs mPGES-1$^{-/-}$ cells cultured in 10% FBS (48 h). Table with subset of up- (red) and down- (green) regulated miRNAs in mPGES-1$^{+/+}$ vs mPGES-1$^{-/-}$ cells. (**B**) Dicer and Drosha protein expression in nuclear (top panel) and cytosolic (bottom panel) extract from DU145 mPGES-1$^{+/+}$ and mPGES-1$^{-/-}$ cells maintained in 10% FBS for 18 h. β-tubulin and lamin (for nuclear extract) were used to normalize loading. *N* = 3. (**C**) Western blot of AP1 (cFOS and cJUN) protein expression in nuclear extract from DU145 mPGES-1$^{+/+}$ and mPGES-1$^{-/-}$ cells maintained in 10% FBS for 18 h. Lamin was used to normalize loading. *N* = 3.

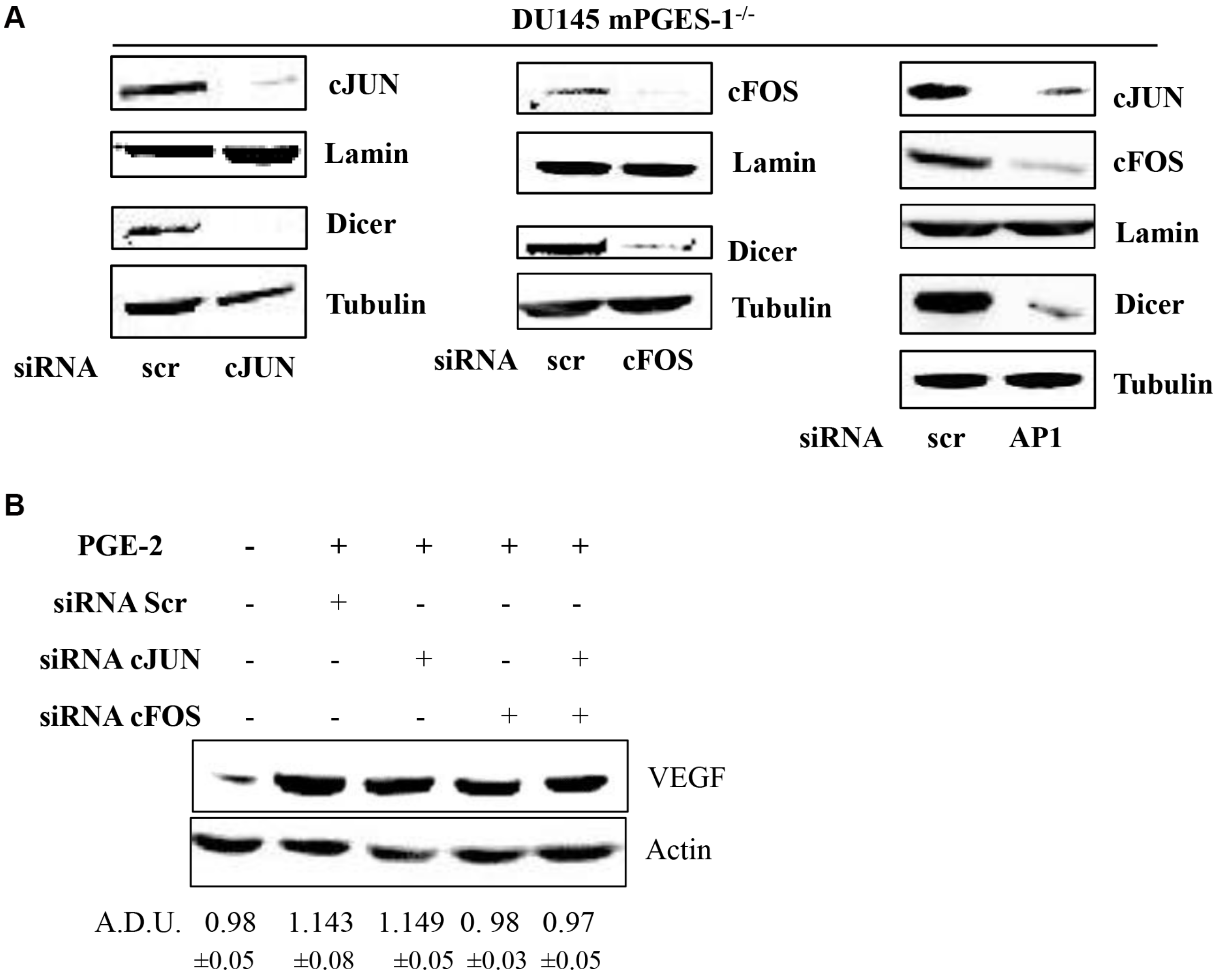

Supplementary Figure S3: AP1, Dicer and VEGF expression in AP1 silenced cells. (**A**) cJUN, cFOS and Dicer protein expression in nuclear and cytosolic extract, of transfected DU145 mPGES-1$^{-/-}$ cells for scramble, cJUN, cFOS and for cJUN+cFOS siRNA, respectively. β-tubulin and lamin were used to normalize loading. $N = 3$. (**B**) VEGF protein expression of scrambled, cJUN, cFOS and cJUN+cFOS siRNA-transfected DU145 mPGES-1$^{-/-}$ cells exposed to PGE-2 (24 h). b-actin was used to normalize loading. $N = 3$. Results are expressed in A.D.U.

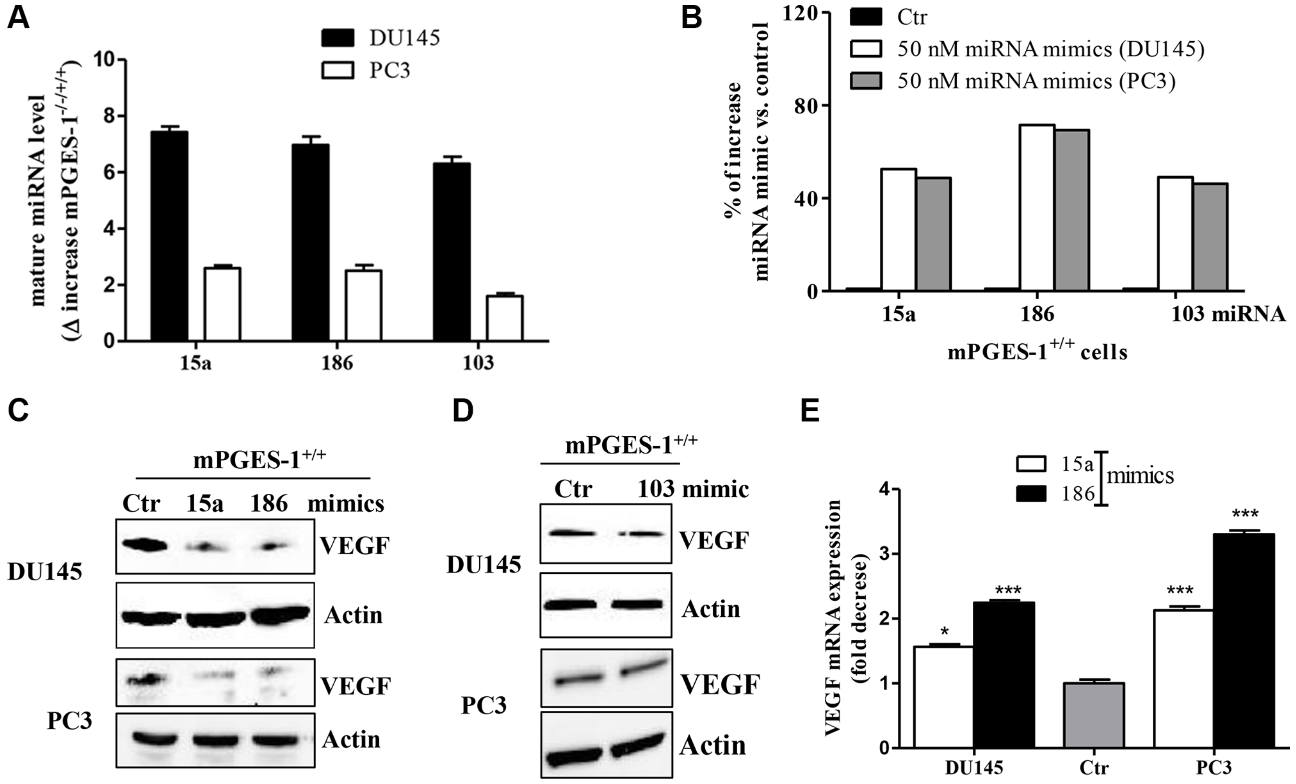

Supplementary Figure S4: 15a and 186 miRNA mimics decrease VEGF expression in DU145 and PC3 mPGES-1$^{+/+}$. (**A**) Δ increase in mature miRNA (15a, 186 and 103) in DU145 and PC3 mPGES-1$^{-/-}$ compared to mPGES-1$^{+/+}$ cells maintained in 10% FBS for 48 h. (**B**) % increase in miRNA in DU145 and PC3 mPGES-1$^{+/+}$ cells after treatment with 50 nM of miRNA mimics. VEGF protein expression in DU145 or PC3 mPGES-1$^{+/+}$ cells (10% FBS, 48 h) transfected with miR-15a, 186 (**C**) or 103 (**D**) mimics (50 nM). β-actin was used to normalize loading. $N = 3$. (**E**) VEGF mRNA expression in DU145 and PC3 mPGES-1$^{+/+}$ (10% FBS, 48 h) transfected with miR-15a, or 186 mimics (50 nM). Data are reported as fold decrease of mPGES-1$^{+/+}$ vs. mPGES-1$^{+/+}$ control cells. *P <0.05; ***P <0.001 vs. mPGES-1$^{+/+}$ control cells.

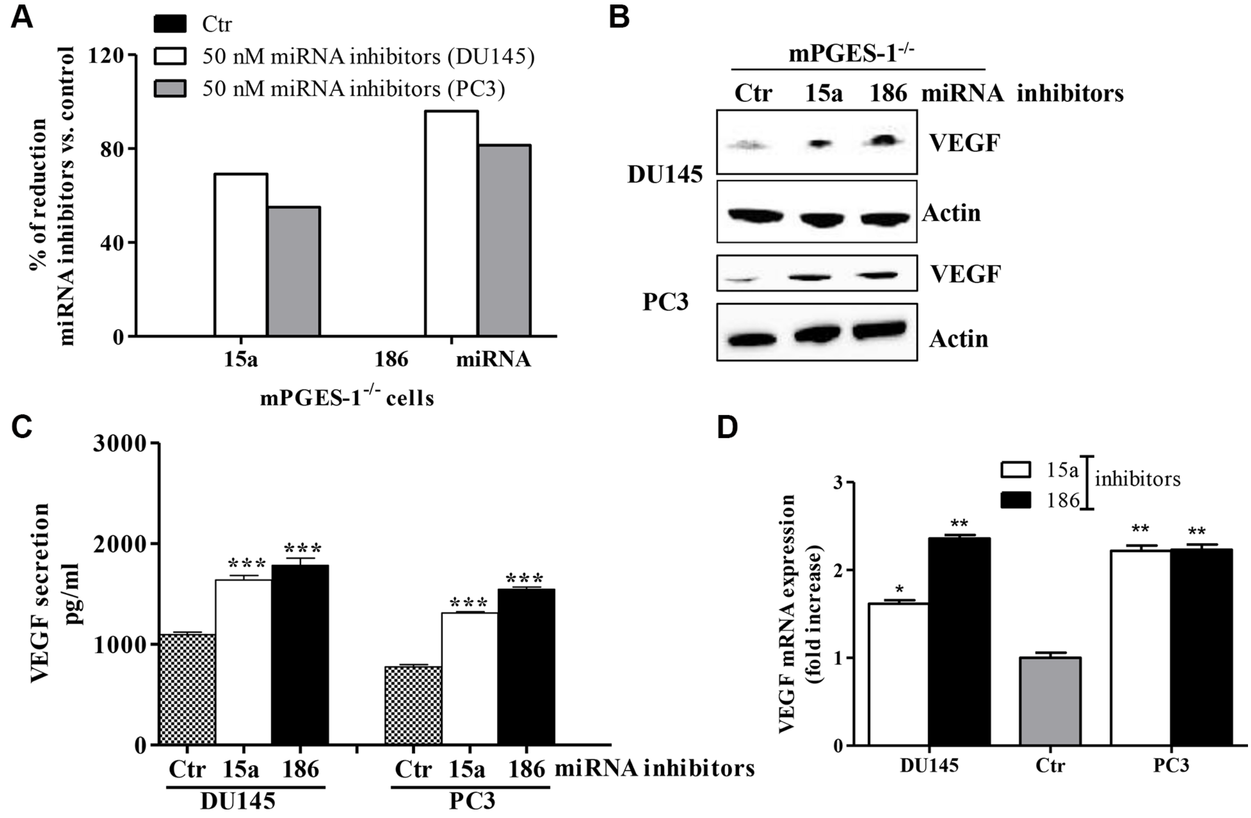

Supplementary Figure S5: 15a and 186 miRNA inhibitors increase VEGF expression/production in DU145/PC3 mPGES-1$^{-/-}$ cells. (**A**) % reduction of miRNA in mPGES-1$^{-/-}$ cells after treatment with 50 nM (alone) of miRNA inhibitors. (**B**) VEGF protein expression in DU145 or PC3 mPGES-1$^{-/-}$ cells (10% FBS, 48 h) transfected with miR-15a or miR-186 inhibitors (50 nM). β-actin was used to normalize loading. *N* = 3. (**C**) ELISA immunoassay for VEGF in DU145 or PC3 mPGES-1$^{-/-}$ cells maintained in 1% FBS for 48 h after transfection with 50 nM miR-15a or miR186 inhibitors. ****P* < 0.001 compared to mPGES-1$^{-/-}$ cells. (**D**) VEGF mRNA expression in DU145 and PC3 mPGES-1$^{-/-}$ cells (10% FBS, 48 h) transfected with miR-15a, or 186 inhibitors (50 nM). Data are reported as fold increase of mPGES-1$^{-/-}$ cells vs. mPGES-1$^{-/-}$ control cells. **P* < 0.05; ***P* < 0.01 vs. mPGES-1$^{-/-}$ control cells.

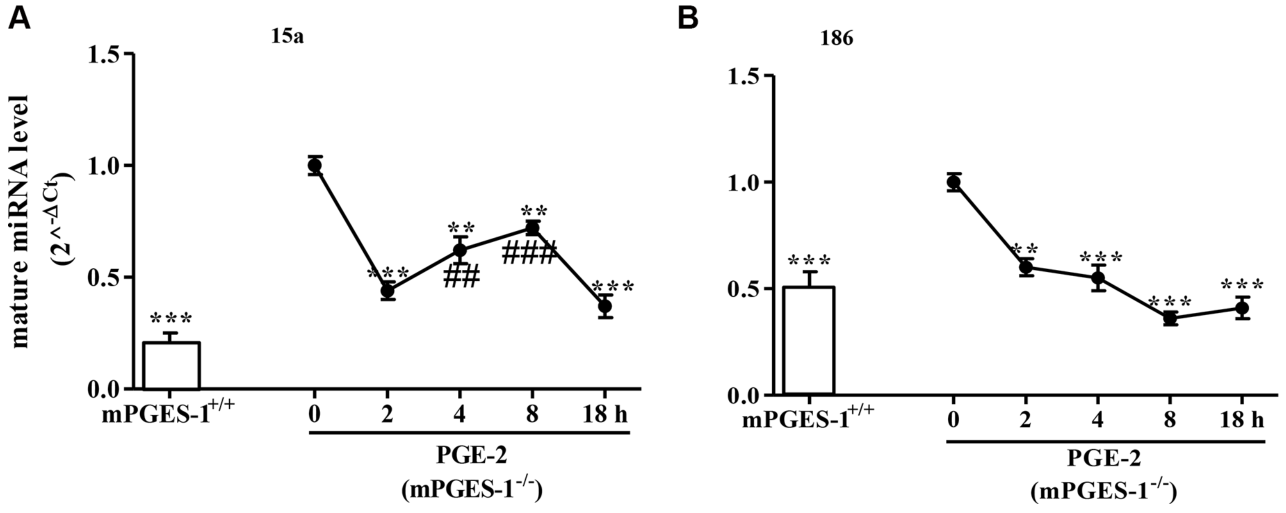

Supplementary Figure S6: PGE-2 administration decreases 15a and 186 miRNA expression in DU145 mPGES-1$^{-/-}$ cells. mRNA levels of mature miRNA 15a (**A**) or 186 (**B**), measured by QPCR in mPGES-1$^{+/+}$ cells cultured in 10% FBS for 48 h or in mPGES-1$^{-/-}$ exposed to PGE-2 (1 μM) for the indicated times. Results are expressed as $2^{\wedge-\Delta Ct}$. ****P* < 0.001, ***P* < 0.01 compared to mPGES-1$^{-/-}$ cells in basal condition. $^{\#\#}$*P* < 0.01, $^{\#\#\#}$*P* < 0.001 compared to mPGES-1-/- cells plus PGE-2 at 18 h.

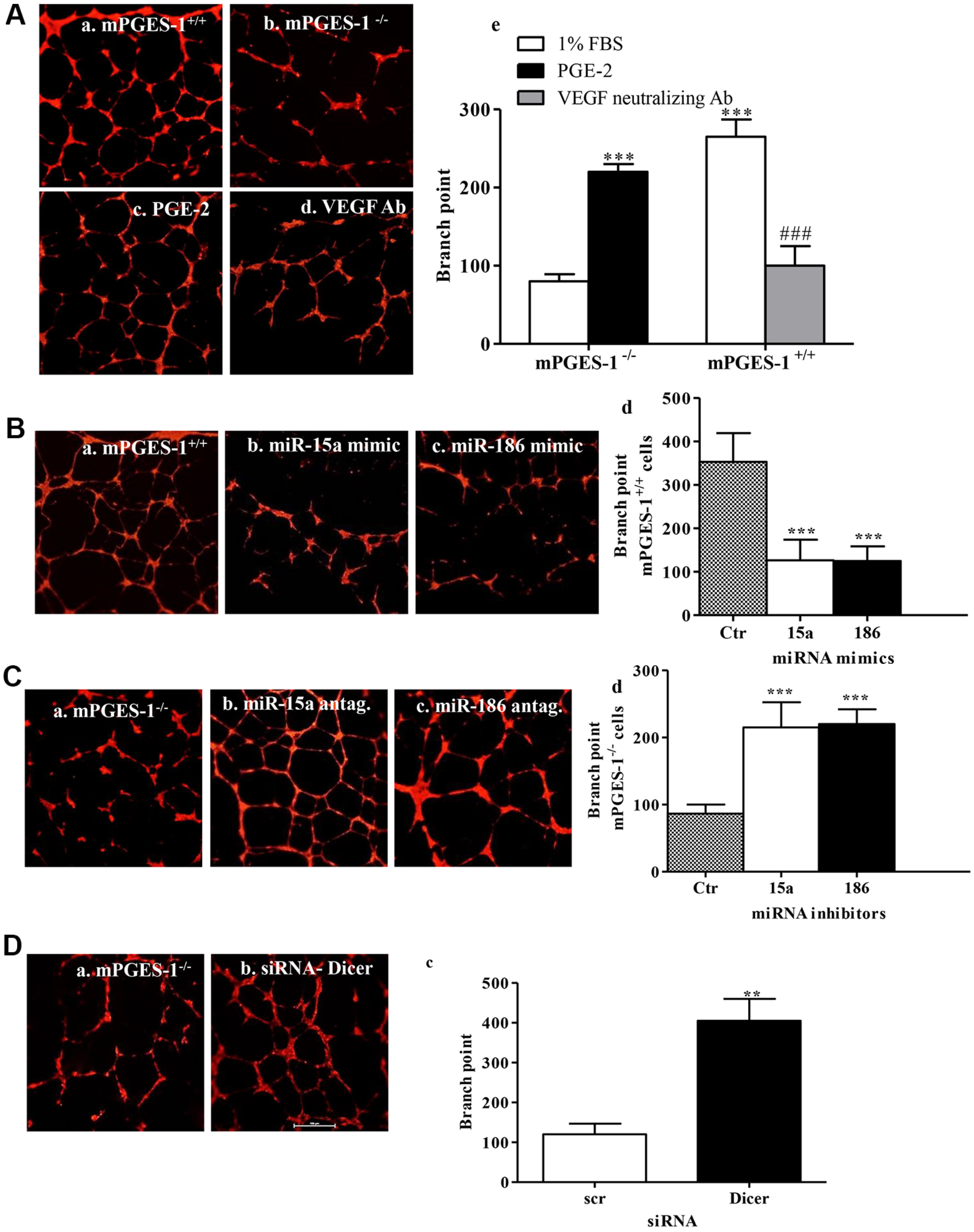

Supplementary Figure S7: Endothelial cell sprouting in co-culture with in DU145 mPGES-1$^{+/+}$ cells or DU145 mPGES-1$^{-/-}$. Images of cord-like network formation in Matrigel by HUVEC co-cultured with: (**A**) mPGES-1$^{+/+}$ cells (A), mPGES-1$^{-/-}$ cells (**B**), mPGES-1$^{-/-}$ cells pre-treated with PGE-2 (1 µM, **C**), or mPGES-1$^{+/+}$ cells pre-treated with VEGF neutralizing antibody (250 ng/ml, **D**). (**E**) Quantification of sprouting performed by counting the number of branch points per well. *N* = 3 ****P* < 0.001 vs. mPGES-1$^{-/-}$ cells, $^{###}p$ < 0.001 vs. mPGES-1$^{+/+}$ cells; (B) mPGES-1$^{+/+}$ cells in control condition (A), or transfected with miR-15a (50 nM, B) or miR-186 mimics (50 nM, C) in 10% FBS for 48 h. (D) Quantification of sprouting performed by counting the number of branch points per well. *N* = 3. ****P* < 0.001 vs. mPGES-1$^{+/+}$ cells in basal condition; (C) mPGES-1$^{-/-}$ cells in control condition (A), or transfected with miR-15a (50 nM, B) or miR-186 inhibitors (50 nM, c) in 10% FBS for 48 h. (D) Quantification of sprouting performed by counting the number of branch points per well. *N* = 3. ****P* < 0.001 vs. mPGES-1$^{-/-}$ cells in basal condition; (D) DU145 mPGES-1$^{-/-}$ exposed to scrambled (A) or to Dicer siRNA-transfected cells (B). (C) Quantification of sprouting performed by counting the number of branch points per well. *N* = 3. ***P* < 0.01 compared to scrambled siRNA-transfected cells.

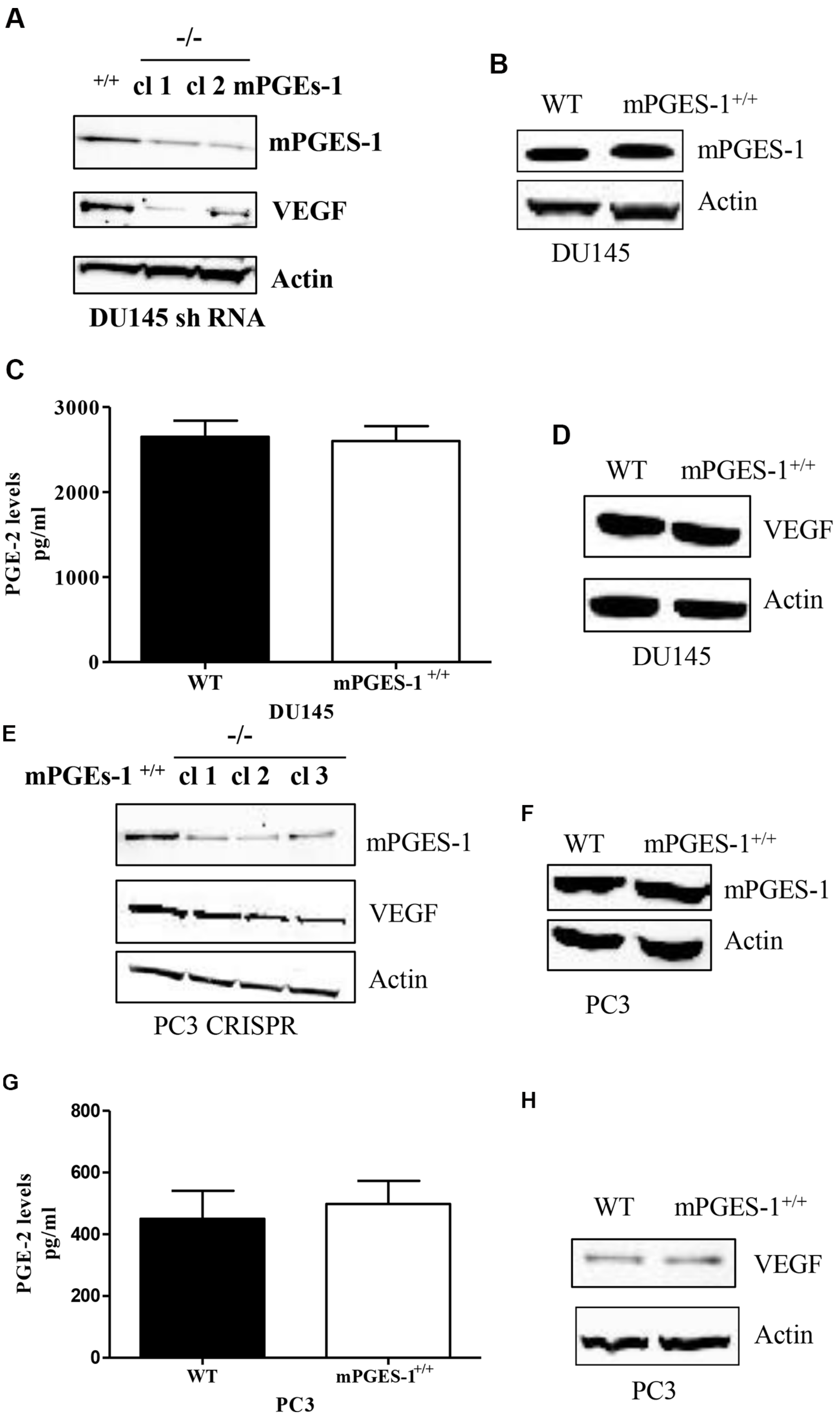

Supplementary Figure S8: Expression of mPGES-1/PGE-2 and VEGF in DU145 and PC3 WT and mPGES-1$^{+/+}$. (A) Western blot analysis of VEGF and mPGES-1 expression in DU145 mPGES-1+/+ and in two different clones of mPGES-1-/- cells exposed to 10% FBS (48 h). β-actin was used to normalize loading; *N* = 3 (**A**–**B**) Western blot analysis of mPGES-1 protein expression in DU145 WT and mPGES-1$^{+/+}$ cells exposed to 10% FBS for 48 h. β-actin was used to normalize loading. *N* = 3. (B–C) EIA immunoassay for PGE-2 in DU145 WT and mPGES-1$^{+/+}$ cells maintained 48 h in 1% FBS. (**C**–**D**) Western blot analysis of VEGF protein expression in DU145 WT and mPGES-1$^{+/+}$ cells exposed to 10% FBS for 48 h. β-actin was used to normalize loading. *N* = 3. (**E**) Western blot analysis of VEGF and mPGES-1 expression in PC3 mPGES-1+/+ and in three different clones of mPGES-1-/- cells exposed to 10% FBS (48 h). β-actin was used to normalize loading; *N* = 3 (D–F) Western blot analysis of mPGES-1 protein expression in PC3 WT and mPGES-1$^{+/+}$ cells exposed to 10% FBS for 48 h. b-actin was used to normalize loading. *N* = 3. (E–G) EIA immunoassay for PGE-2 in DU145 WT and mPGES-1$^{+/+}$ cells maintained in 1% FBS for 48 h. (**F**–**H**) Western blot analysis of VEGF protein expression in PC3 WT and mPGES-1$^{+/+}$ cells exposed to 10% FBS for 48 h. b-actin was used to normalize loading. *N* = 3.

Supplementary Table S1: Principal functions of miRNA modulated in our array

miRNA	Fold change	*P* value	Angiogenesis	Plurypotency	Epidermal Differentiation	Survival	Cell Cycle
miR-29b	–4,97	< 0.001	X				
let-7a	–2,73	< 0.05		X			
let-7b	–3,72	< 0,01		X	X		
miR-181a	–5,31	< 0.001					
miR-29a	–3.55	< 0.01	X				
miR-22	–3.79	< 0.01		X			
miR-141	–7.31	< 0.001		X			
miR-92a	–2.18	< 0.05	X				
miR-130a	–5.23	< 0.001	X	X			
let-7i	–2.83	< 0.05					
miR-93	–2.73	< 0.05	X				
let-7g	–3	< 0.01		X			
miR-15b	–2.56	< 0.05	X				
miR-186	–3.39	< 0.01	X				
miR-29c	–3.43	< 0.01	X				
let-7d	–4.18	< 0.001					
miR-15a	–3.86	< 0.001					X
miR-196b	–2.65	< 0.05					
miR-103	–3.14	< 0.01	X				
miR-222	–5.27	< 0.001	X	X			
miR-210	–10.36	< 0.001			X		
miR-125b	2.68	< 0.05	X			X	
miR-376	2.57	< 0.05	X			X	

The miRNAs up- and down-regulated in mPGES-1$^{-/-}$ cells (2.5 fold) are reported to be involved in several functions.

Supplementary Table S2: Sequences of miRNA, up- and down-regulated in our array, in the 3′ UTR region of pro-angiogenic factors (VEGF, HIF-1α)

	Mature miRNA sequence
Hs-miR-15a	5′-UAGCAGCACAUAAUGGUUUGUG-3′
Hs-miR-15b	5′-UAGCAGCACAUCAUGGUUUA-3′
Hs-miR-93	5′- CAAAGUGCUGUUCGUGCAGGUAG.3′
Hs-miR-103	5′-AGCAGCAUUGUACAGGGCUAUGA-3′
Hs-miR-186	5′-CAAAGAAUUCUCCUUUUGGGCU-3′

Supplementary Table S3: Expression of mPGES-1, VEGF, HIF-1α and Dicer in donors of prostate tissues analyzed in Figure 7

PCa	mPGES-1 + VEGF + HIF-1α + Dicer –	mPGES-1 – VEGF – HIF-1α – Dicer +	mPGES-1 – VEGF – HIF-1α – Dicer –	mPGES-1 + VEGF + HIF-1α + Dicer +
OC	5 (20%)	17 (68%)	1 (4%)	2 (8%)
AC	18 (66.7%)	6 (22.2%)	0	3 (11.1%)
